# Pericytes: Biomarkers and Roles in Thoracic Aortic Aneurysm

**DOI:** 10.3390/genes17050555

**Published:** 2026-05-05

**Authors:** Theodora M. Stougiannou, Dimos Karangelis

**Affiliations:** Department of Cardiothoracic Surgery, University General Hospital, Democritus University of Thrace, 68132 Alexandroupolis, Greece; theodorastougiannos@gmail.com

**Keywords:** cardiovascular, biology, physiology, aorta, adventitial, pericyte, genes, thoracic aortic, aneurysm

## Abstract

The aorta is the largest vascular conduit in humans, comprising three layers and a multitude of varying cell types collectively maintaining homeostasis and normal aortic wall function. Amongst these layers, the tunica adventitia is the external-most layer, where microvessels, termed vasa vasorum, can be found. These comprise pericytes and endothelial cells (ECs) and provide nourishment to the tunica adventitia and the outer media layers in the thoracic aorta. Adjacent to these microvessels, stem/progenitor group populations can be found, together forming a perivascular niche. Eventually, however, many of these cells and components can become dysregulated and contribute to development of thoracic aortic aneurysm (TAA). The purpose of this narrative review is to evaluate the recent literature related to marker gene expression in tunica adventitia pericytes, as well as the contribution of these populations to the development of aneurysm in the thoracic aorta. Pericytes in TAA generally exhibit phenotypic changes, which could be driven, in part, by loss of fibroblast growth factor (FGF) signaling. These changes eventually lead to vasa vasorum remodeling in the thoracic aorta, in turn contributing to the development of TAA.

## 1. Introduction

Pericytes are mural cells with stellate morphology, often seen enveloping smaller blood vessels. They are identified by their close proximity to endothelial cells (ECs), usually sharing a basement membrane; they are also characterized by specific gene expression, characteristics and behavior, both in vivo and in vitro [1]. Due to the widespread localization of pericytes across different vascular beds, they contribute to a wide range of pathologies, including those that may affect the physiological function of the vascular wall. The contribution of pericytes to arterial wall calcification in atherosclerosis was identified for the first time in 1993, with identification of pericyte–like cells expressing the 3G5 antigen, a surface ganglioside normally identified in pericytes, within the calcified nodules of the atherosclerotic aortic wall [2]. Ever since, many more studies have evaluated the role of pericytes in the development of aortic atherosclerosis [3], as well as their capacity for multipotent lineage generation [1,4]. The contribution of genetic and epigenetic factors to the development of aortic pathology has been extensively studied, highlighting the importance of identifying new biomarkers that contribute to the emergence and propagation of aortic pathology, including aortic aneurysm [5,6]. Involvement of pericytes in these processes, however, has only just more recently been explored [7,8].

The purpose of this narrative review is to summarize and evaluate information related to pericyte populations in the aortic wall. This includes cellular characteristics and behavior, marker expression differentiating pericytes from other populations in the area, and pericyte plasticity, as well as the extent of involvement in thoracic aortic aneurysm (TAA). With respect to the latter, this text aims to summarize and discuss recent findings in relation to the contribution of pericytes to the progression of TAA. These findings will be then framed in the context of pericyte characteristics and behavior, as well as plasticity in the tunica adventitia of the aortic wall. A review of the literature has been carried out across PubMed, ScienceDirect and Google Scholar, with dates spanning approximately 1979, the first mention of pericytes in association with the aortic wall in the literature, up to 2025. The keywords used include (pericytes OR mural) AND (aorta OR aortic wall OR vascular wall), (pericytes) AND (thoracic aorta) AND (disease OR aneurysm), (pericytes) AND (aorta OR aortic wall) AND (development). Only studies evaluating pericytes present in the aortic wall have been included in this text, to more specifically frame the findings of recent studies to the general characteristics and function of these cell populations.

## 2. Pericytes

Described as mural cells, pericytes are found in the vascular wall in close association and interact with ECs. Through these interactions they contribute to the overall physiology and, oftentimes, pathophysiology of the vascular wall [9]. Pericytes have been observed in various microvessel types (capillaries, post–capillary venules, and terminal arterioles) found throughout body systems, as well as larger vessels [10]. In the latter, stellate pericyte–like cells have been found in the subendothelial layer alongside other cell groups such as intimal fibroblasts, vascular smooth muscle cells (SMCs) and immune cell populations. This layer, found in larger vessels as well, is identified between the basement membrane and the internal elastic lamina (IEL) [3,11]. Pericytes can also be found in the adventitia of these larger vessels, in association with microvessels in the tunica adventitia [1,4]. With regards to cellular morphology, pericytes have a large cell body with a relatively large nucleus and small cytoplasm. They also have several cytoplasmic processes, which can be seen extending to and surrounding nearby microvessels. A single pericyte can be often seen incompletely enveloping multiple ECs. In turn, these pericyte extensions can be either circumferential or be present longitudinally along the length of the vessel [12,13]. The amount of EC and pericyte coverage depends on the tissue. For example, in the central nervous system (CNS) and retina, there is a pericyte–to–endothelial ratio of ~1:1 to ~1:4 and 1:1, respectively, among the highest coverage rates observed, to ensure barrier integrity. In other areas, however, such as skeletal muscle, this ratio falls to approximately ~1:10 to 1:100, though this can vary depending on tissue. The reduced pericyte coverage in most peripheral tissues reflects the reduced need for rigorous barrier requirements in these locations [14]. Regarding biomarker signature, studies evaluating pericyte expression across varying organ systems (skeletal muscle, placenta, pancreas, adipose tissue, heart, intestine, bone marrow, lung, and CNS) generally describe the gene markers CD146+/Neural glial antigen 2 (NG2+)/platelet–derived growth factor receptor beta (PDGFRβ)+/alkaline phosphatase (ALP)+/CD34–/CD45– as the universal pericyte marker expression phenotype [4].

**Figure 1:**
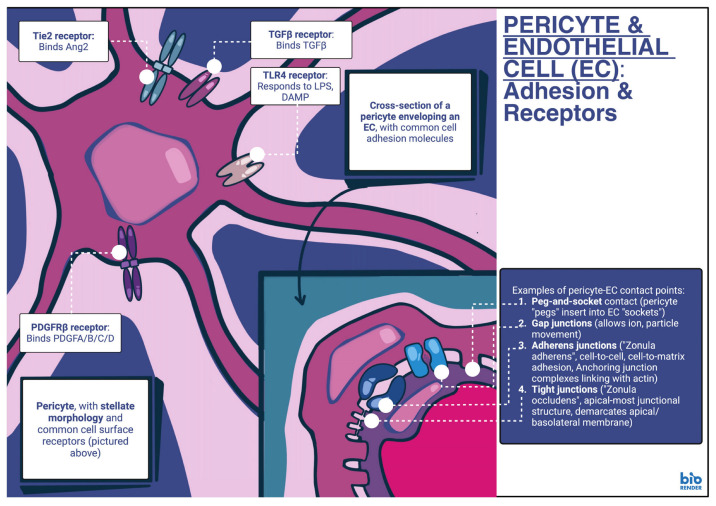
Pericyte and endothelial cell (EC). A pericyte is shown along with an adjacent endothelial cell (EC) in the lower right corner; the stellate morphology of the pericyte is shown on the lefthand side of the illustration. Additional aspects pictured include the intercellular connections with adjacent ECs and some indicative protein markers associated with pericytes and ECs, respectively. While all these markers [Tie2, TLR4, TGFβ receptor II, PDGFRβ] are found in both pericytes and ECs, for some, cognate binding molecules can differ. For example, while Tie2 is expressed in both pericytes and EC, pericyte Tie2 mainly binds EC–derived angiopoietin 2 and is commonly involved in vascular destabilization. On the other hand, EC Tie2 binds pericyte–derived angiopoietin 1 and is mainly involved in vascular maturation [20]. Ang1/2, angiopoietin 1/2; DAMP, danger-associated molecular patterns; EC, endothelial cell; LPS, lipopolysaccharide; Tie2, TEK receptor tyrosine kinase (TEK); TGFβ/1, transforming growth factor beta/1; TLR4, Toll-like receptor 4; PDGF–A/B/C/D, platelet–derived growth factor A/B/C/D; PDGFRβ, Platelet growth factor receptor beta. [Illustration created digitally with the painting application ‘Procreate^®^’; composite graphic assembled with BioRender.com. (Created in BioRender. Stougiannou, T. (2026) https://BioRender.com/7apgnmc, accessed on 16 March 2026)].

Pericytes usually interact with ECs, as they both share a common basement membrane. Furthermore, both ECs and pericytes contribute to the production of ECM components in this basement membrane [14]. Pericytes are usually positioned approximately ~20 nm away from ECs and interact with multiple ECs, at specific, discrete points. These points can include peg-and-socket contacts, for example, where pericyte cytoplasmic fingers, also known as pegs, insert into EC invaginations (pockets). Peg-and-socket contacts comprise junctional proteins, including N-cadherin and connexin 43. They contribute to both mechanical anchorage as well as functional connection between the two cell types. Gap junctions are channels that facilitate signal transduction via intercellular movement of small molecules and ions/ionic currents. Structural proteins such a connexin 43 can be identified here as well [15]. Adherens junctions allow for linking between cytoskeleton components of adjacent pericytes and ECs, forming adhesion plaques. These adhesion plaques in turn contribute to the mechanical anchorage between these two cell types; they comprise proteins such as N-cadherin and fibronectin [15]. Fibronectin, in particular, contributes to the stability of the junction [16]. Finally, tight junctions allow for barrier formation in locations such as the blood–brain barrier (BBB) [17] as well as the blood–retina barrier (BRB) [18]. They are usually composed of protein components such as occludin, claudin–12, and zona occludens–1/2. All in all, these cell adhesion complexes are generally involved in vessel maturation and stability, regulation of vascular permeability, angiogenesis and vascular remodeling. In addition, they regulate responses to physiologic as well as pathophysiologic stimuli [18,19] (Figure 1).

## 3. Aorta

### 3.1. Brief Overview of Structure

The aorta is composed of three layers, the tunica intima, tunica media and tunica adventitia. The tunica intima is the innermost section, comprising a single endothelial layer, the basement membrane, and, usually, a subendothelial layer as well. The IEL, composed of elastic fibers, abuts the tunica intima. Following is the tunica media, composed of vascular SMC layers interspersed with ECM proteins, the principal components of which are collagen and elastin. Lamellar units, or layers, are units of elastin fibers surrounding vascular SMC tissue. Usually, the number of lamellar layers (units) present is proportional to the wall pressure that develops in each aortic segment; the number of lamellar layers also differs between segments. The external elastic lamina (EEL), a layer of elastic fibers abutting the tunica media, can be identified here as well. This is the layer mainly responsible for the mechanical attributes of the aortic wall [21,22]. Finally, the tunica adventitia is the external-most layer of the aorta and it is mainly composed of collagen type I and fibroblasts, alongside other cellular populations. These populations will be described in greater detail in subsequent sections [23].

Though the general structure remains unchanged along its length, variations occur, which, in turn, account for differential requirements in optimal wall pressure and stress in different aortic segments. The thoracic aorta in humans generally includes the aortic root (base of the aortic leaflet to the sinotubular junction), ascending aorta (sinotubular junction to the aortic arch), aortic arch (curved segment between ascending aorta and descending aorta) and descending thoracic aorta (aortic arch to diaphragm). The aortic root and ascending aorta have a higher elastin content, as well as thicker tunica media, allowing for distension and subsequent recoil during each cardiac cycle, as these segments receive the cardiac ejection fraction [23]. This converts pulsatile cardiac flow into continuous blood flow, which will then flow throughout the vascular system, described as the ‘Windkessel effect’ [24]. Along the length of the aorta, elastin fiber content and tunica media thickness decrease, with the infrarenal abdominal aorta possessing low levels of elastin with reduced wall thickness. On the other hand, tunica adventitia thickness and collagen content in the aortic wall increase along its length, with highest collagen levels and tunica adventitia thickness reported for the abdominal aorta. As a result, the elastin-to-collagen ratio is higher in the ascending thoracic aorta compared to other sections. This renders aortic segments in locations with lower elastin-to-collagen ratio less compliant and more prone to mechanical injury [23,25,26,27].

### 3.2. Adventitia and the Vasa Vasorum Network

The adventitia is the outermost layer of the vascular wall, comprising loose connective tissue, vasa and nervi vasorum, and tissue resident inflammatory cells, as well as stem/progenitor cell populations. The adventitial ECM is composed of collagen fibers embedded within a ground matrix, preventing overexpansion at high pressures. As already stated, along the length of the aorta, vascular wall thickness and tunica media thickness decrease overall, whilst on the other hand, tunica adventitia thickness increases, at least in animal models [23,25,26,27]. More specifically, in the ascending thoracic aorta, tunica adventitia thickness is greatest at the level of the aortic root and thinner at level of the pericardium [21,28].

A fourth layer can also be recognized, usually in arteries and veins with a diameter greater than 100 μm. This layer is known as perivascular adipose tissue (PVAT) and is defined as a depot of adipose tissue external to the adventitia, though no clear anatomical boundary between the two usually exists. PVAT can be identified in various arteries, including the aorta and the coronary, mesenteric, femoral and renal arteries [29]. Conversely, no PVAT is found in cerebral and pulmonary vasculature [30]. In the thoracic aorta of mice, PVAT exhibits characteristics of brown adipose tissue (BAT), although in humans, PVAT can more accurately be described as BAT–like. In the latter, adipocytes are smaller and morphologically similar to white adipose tissue (WAT) adipocytes. Regardless of these characteristics, however, these adipocytes exhibit expression of BAT cell markers [29]. In the ascending thoracic aorta of mice, PVAT comprises mainly of BAT and originates from the neural crest. Similarly, in humans, as with PVAT in the thoracic aorta in general, adipose tissue in this location exhibits BAT–like characteristics despite the smaller adipocyte size. This includes expression of BAT proteins such as Uncoupling protein–1 or thermogenin [UCP–1] and Peroxisome proliferator-activated receptor gamma coactivator 1–alpha [PGC–1α]) [31]. The PVAT provides metabolites, including vasoactive molecules, adipokines and various inflammatory cytokines. It is also involved in both physiological and pathophysiological processes of the aortic wall [30,31,32]. As a result, an adventitia–periadventitial functional unit is formed, composed of the cellular populations present in the tunica adventitia, including stem/progenitor cell groups, along with microvessels, nerves, lymphatics and PVAT [33].

As previously mentioned, the vasa vasorum comprise a vascular network within the tunica adventitia. From their location in the tunica adventitia, they usually extend into the PVAT, as well as the outer layer of the tunica media. This latter phenomenon is often dependent on the thickness of the vascular wall as well as the number of lamellar layers present. In arteries with a wall thickness less than 0.5 mm, or, alternatively, arteries that possess less than 29 lamellar layers, vasa vasorum do not extend into the tunica media observed [22]. Conversely, vasa vasorum extension into the tunica media increases in range with age, possibly to account for the reduced capacity for nutrient diffusion from the luminal aspect of the vessel [28]. Overall, the ascending thoracic aorta possesses a denser vasa vasorum network, one which extends deeper into the inner segments, at least compared to other aortic sections. This is required due to the increased wall thickness, tunica media thickness and greater elastin content of the thoracic aorta compared to other segments. As a result, the thoracic aorta contains 25 vascularized lamellar units and another 35 units that are avascular. On the contrary, less than 28 lamellar units occur in the abdominal aorta, compared to the thoracic aorta [34]. There is also reduced vasa vasorum density in the infrarenal abdominal aorta [28].

Based on micro–CT studies of aortic tissue specimens, vasa vasorum can be generally classified into first-order vasa vasorum, identified longitudinally along the arterial wall with a size comparable to that of arteriolar vessels, and second-order vasa vasorum. The latter are identified circumferentially around the arterial wall with a size comparable to that of capillaries [1,35]. In the ascending thoracic aorta, first-order vasa vasorum originate from brachiocephalic and coronary arteries, while in the descending thoracic aorta, first-order vasa vasorum usually originate from intercostal arteries [22]. In general, the density of the vasa vasorum in the aortic wall may vary depending on life stage (reduction in density with age), as well as pathophysiology. For example, aneurysm of the ascending thoracic aorta is associated with reduced vasa vasorum density in the adventitia [35,36].

Vasa vasorum in the tunica adventitia generally include arterioles, capillaries and post-capillary venules. They contribute to the cycling and transportation of cells as well as biologically active factors, from the abluminal towards the luminal layers [37]. Many different cell types can be identified in the adventitia of the aorta. Fibroblasts are the principal cellular type in both the ascending thoracic as well as the abdominal aorta. They generate collagen type I and II, along with elastin (albeit in smaller amounts compared to the tunica media), and, along with the proteoglycans present, contribute to the ECM composition of the vascular wall [38]. Fibroblasts in the tunica adventitia can also be induced by urotensin II, a vasoconstrictor, to generate osteopontin. Urotensin II, in turn, can be upregulated in several pathological cardiovascular conditions, including atherosclerosis and essential hypertension [39], coronary artery disease [40] and congestive heart failure [41]. Osteopontin will then induce migration and phenotypic conversion of adventitial fibroblasts, contributing to vascular remodeling [42]. Additional cell types in the adventitia include myofibroblasts involved in the remodeling of the vascular wall, various immune cell populations (macrophages, dendritic cells, T–lymphocytes, B–lymphocytes, and mast cells) and adipocytes [37,43]. Comprising the vasa vasorum, EC and pericyte cell populations can be observed as well, along with adjacent stem/progenitor cell populations. In the ascending thoracic aorta, the adventitial vasa vasorum network is especially dense. This, in association with the perivascular cell populations present, contribute to its characterization as a specialized perivascular niche [1].

## 4. Aorta and Cell Populations

### 4.1. Tunica Adventitia and Cell Populations

As already described in previous sections, the adventitial vasa vasorum in the ascending thoracic aorta form a rich vascular bed, and, along with adjacent populations, assemble into a perivascular niche. Various populations have been identified in this location via flow cytometry and have been collectively described as adventitial cells. Populations of pericytes mainly expressing biomarkers such as CD146+/actin alpha 2, smooth muscle (ACTA2) (α–SMA) ±/NG2+/CD34−/von Willebrand factor (vWF)− are seen surrounding ECs. These EC populations, in turn, are characterized by the biomarker signature α–SMA−/CD34±/CD31+/vWF+ [1]. CD31, in particular, is a marker associated with mature EC groups, while CD105, normally associated with mesenchymal stromal cells (MSCs), can be identified in both pericytes and ECs of the perivascular niche [1]. MSCs are generally defined as a bulk population with unique secretory, immunomodulatory and homing abilities, while mesenchymal stem cells, on the other hand, have a well-demonstrated function as progenitor populations, capable of self-renewal and differentiation. As per the guidelines put forth by the International Society for Cell & Gene Therapy (ISCT^®^), multipotent MSCs can be defined by a specific set of minimal criteria, including plastic adherence, expression of markers, such as CD73, CD90, and CD105, and lack of gene expression that denotes hematopoietic or endothelial lineage. As such, these cells lack CD11b, CD14, CD19, CD34 (though the absence of CD34 for the definition is not absolute), CD45, CD79a and HLA–DR. Finally, these cell groups possess capabilities for differentiation into adipocyte, chondrocyte and osteoblast progeny populations [44].

Additional populations can be observed superficially to the vasa vasorum of the niche and have been described as supra–vasa or adventitial cells, owing to this location. These cells are of both non–endothelial (CD31–) and non–pericytic (CD146–) origin, possess the gene marker expression profile of CD34+/CD31–/CD146, and exhibit potential for differentiation into bone, smooth muscle, cartilage, adipose, pericyte and endothelial lineages [1]. Owing to these characteristics and potential, these populations could be regarded as adipose stem cell (ASC) populations. Regarding the EC populations identified in the perivascular niche of the ascending thoracic aorta, mature endothelial populations expressing CD31+ can be identified, although some subsets express CD34+ as well. There is a relatively high prevalence of mature EC groups in these tissues, which is to be expected, owing to the high prevalence of vasa vasorum networks in the area [1]. Despite this, such cells, though developmentally mature, still have the potential for Endothelial–to–mesenchymal transition (EndMT) [45], as well as adipose tissue differentiation [46]. Furthermore, analyses of the perivascular niche in adipose tissue non–specific to the aortic wall reveal spatial restriction of stem/progenitor populations. More specifically, in these locations, progenitors expressing CD201 are restricted to the outer adventitial layers. Cells with high CD201 expression represent primitive mesenchymal stem/progenitor populations, while cells with reduced CD201 expression correspond to more committed mesenchymal progenitors. The latter also possess increased differentiation potential towards osteogenic and adipogenic lineages. Similar perivascular niche locations have been identified within adipose tissue of various arteries and veins; in some studies, these areas range from approximately 450 to 1100 μm in diameter. In these locations, CD201 often colocalizes with CD34, representing adventitial cells characterized by CD34+/CD31–/CD45–. Around 5.4% of these cells express high levels of CD201 as well [47].

Endothelial progenitor cells (EPCs) can also be found in the perivascular niche, though these may originate from the circulation. EPCs express CD31 and CD34, along with CD146, a marker classically associated with pericytes; it is usually expressed in EPC groups that also express CD34. Their presence in the perivascular niche could allude to potential mechanisms involved in neovascularization during pathophysiological states [1]. Another marker, CD133, typically absent in mature ECs, is often used to differentiate between mature ECs and EPCs, as it is often expressed in the latter. Additional markers expressed on the EPC surface include vascular endothelial growth factor receptor 2 (VEGFR2)/Kinase insert domain receptor (KDR)+ [48]. In general, EPCs identified in the tunica intima of vessels can thus be characterized by the biomarker profile CD34+/CD45–/Protein tyrosine kinase receptor Flk–1 (Flk–1)+/CD31+/Stem cell antigen–1 (SCA–1)+/KIT proto–oncogene and receptor tyrosine kinase (c–Kit)+. Similar EPC populations found in the tunica adventitia are usually identified as CD34+/Flk–1+/SCA–1+/c–Kit+ in related studies [49].

### 4.2. Pericytes in the Tunica Adventitia

As expected of the local vasa vasorum network, pericyte populations are also observed in the tunica adventitia of the thoracic aorta. These cells express surface markers such as CD146, characteristic for pericyte groups, and CD90, often associated with pericytes present in vasa vasorum networks. They are also negative for markers such as CD31, a hallmark of endothelial populations, and are usually negative for the gene marker CD34, although a subset of pericytes can also express CD34 as well. Pericytes comprise approximately ~15% of all non–endothelial cells (CD31–) in the perivascular niche of the ascending thoracic aorta and are usually defined as CD146+/CD56−/CD45− (non-hematopoietic) and CD31− (non–endothelial). They lie in close proximity to the vasa vasorum of the niche, encircling the microvessels while sharing the same basement membrane with the ECs [1]. In comparison, in the perivascular niche of human adipose tissue, pericytes have been described to comprise up to 32% of non–endothelial (CD31–) populations [46]. Pericytes in the tunica adventitia display gene marker expression profiles similar to that of pericytes present in arterioles in other locations as well [1]. Amongst the pericyte populations present in the perivascular niche of the ascending thoracic aorta, a specific subpopulation expressing CD34 can be identified. This population has been described by some as a transitional state between adventitial cells (CD34+/CD146–) and pericytes (CD34–/CD146+) and could thus represent pericyte progenitor populations instead [1]. Another protein, often expressed by pericytes also positive for CD34 expression, is α–SMA; it is usually found in the CD34+ pericyte populations, in association with smaller vasa vasorum (diameter <25 μm) [1]. NG2 is yet another protein used to characterize pericytes, usually co-expressed with CD146 on the pericyte surface [4]. Furthermore, Nestin and Wilm’s tumor protein, expressed on pericytes found in the vasa vasorum of abdominal aortic tissue, can be identified in the perivascular niche as well [50]. Perivascular pericytes generally exhibit potential for differentiation towards mesenchymal tissue lineages such as bone, smooth muscle, cartilage, adipose tissue and myocardium. The presence of the CD34 marker in pericyte subpopulations that also express α–SMA denotes this capability for differentiation into smooth muscle lineages, while presence of the markers Nestin and Wilm’s tumor protein usually represents potential for differentiation into various stem/progenitor cell groups [1].

#### 4.2.1. Pericytes and Differentiation Potential

As previously noted, pericytes exhibit multilineage differentiation potential towards various mesodermal lineages, including bone, smooth muscle, cartilage [4], adipose tissue [46] and myocardium [51]. This is a characteristic detected in pericyte populations across tissues, and has been observed in pericytes from muscle tissue, the pancreas, WAT [4], placenta and the perivascular niche found in the ascending thoracic aorta [1]. Pericytes natively express markers associated with stem/progenitor cell populations, particularly mesenchymal stem cells, including CD90 and CD105. Both biomarkers are expressed in both pericytes and ECs of the vasa vasorum. In addition, pericytes are also characterized by expression of the CD73 marker, also normally found in stem/progenitor cell groups [4]. In other similar studies evaluating mesenchymal stromal cells and pericytes, however, the general absence of CD34 and CD31 in the latter differentiates these groups from the multipotent stromal progenitors derived from CD34+ adventitial, also known as supra–vasa, cells [1]. Culturing on Matrigel substrate allows for isolated pericytes to form spheroids and localize around branching EC–derived vascular tubes. This behavior further differentiates pericytes from mesenchymal stem [4] and stromal cell populations, though similarities in marker expression and differentiation potential have earned these cells the characterization of being MSC–like [1]. Some characterize pericytes in the perivascular niche as mesenchymal stem cell precursors precisely due to this shared capability for multilineage differentiation [4,52]. Others, however, based on analyses with FACS–mediated purification, maintain that these two groups are different cell populations [1]. In general, pericytes are thus characterized as a different and distinct cellular population when compared to CD34+ adventitia cells, MSCs derived from CD34+ adventitia cells and mesodermal progenitor cells (MPCs) [1]. MPCs, in particular, are defined as a subpopulation of mesenchymal cells normally isolated from the bone marrow, with a capacity for angiogenesis [53]. Any similarities in expression and differentiation capabilities could thus point to the observation that, across many different populations and tissues, MSC–like gene expression and characteristics can be identified [1].

Pericytes also share similarities in gene expression and differentiation potential with ASC and adipose stromal cell (ADSC) groups [54]. Both of these cell types represent multipotent stem/progenitor cell populations isolated from adipose tissue, although ADSCs often represent the stromal vascular fraction isolated from these locations instead [55]. Adipose stem/progenitor groups can generally be isolated from perivascular locations found in adipose tissues; with regard to the tunica adventitia, adipose progenitors can be isolated from PVAT, including the PVAT surrounding the thoracic aorta. Adipose progenitors isolated from this location usually display a tendency for differentiation into adipose and chondrogenic lineages. However, contrary to mesenchymal stem cells isolated from the bone marrow, they cannot as readily differentiate into osteogenic lineages [54]. Both pericytes and adipose progenitor groups thus display multipotency and can differentiate into mesenchymal tissue groups and lineages [1]; both cell types also display similarities in biomarker expression, including CD34 [1,46]. Furthermore, both can express α–SMA upon induction with transforming growth factor beta 1 (TGFβ1) [1]. In fact, pericytes have been described as the progeny of a subset of adipose progenitor cell groups in some studies [1,56]. However, as with the comparison between mesenchymal progenitor groups and pericytes, adipose progenitors and pericytes differ in the expression of other markers, as the former lack expression of stereotypical pericyte markers (CD146, α–SMA, and CD105) in their native state. In addition, pericytes’ capability for spheroid formation and localization around assembling ECs, as previously mentioned, sets these two populations further apart. More likely, it is adventitia cells identified in the perivascular niche of the tunica adventitia that express CD34 but are negative for CD146 expression which are the most likely candidates to be adipose progenitor groups. This is according to studies evaluating the cellular populations comprising the perivascular niche in the tunica adventitia of the ascending thoracic aorta [1].

#### 4.2.2. Pericytes and Function

Pericytes interact with adjacent ECs, jointly contributing to basement membrane secretion; they can have diverse functions depending on the location of the vascular bed in which they are found. Though pericytes fulfill many diverse roles depending on the organ system, only effects relevant to their presence on the vascular wall of larger vessels, as part of the perivascular niche, will be discussed in this text. Pericyte functions are often related to microvessel behavior, such as the regulation of microvascular tone, diameter, permeability and barrier function, at least in cases where a tight tissue barrier is required. Examples include the BBB in the CNS, and the BRB in the retina. They also contribute to angiogenesis [57], affecting processes such as EC proliferation [58]. The multipotent developmental potential of pericytes further allows for the derivation of differentiated cell progeny, as well as the support of local stem/progenitor cell populations [59]. This is true, since CD146+ pericyte populations, originating from the perivascular niche of fetal bone marrow and adipose tissue, have displayed the capability for cell–to–cell contact with hematopoietic stem/progenitor cells ex vivo. This, in turn, leads to activation of Notch signaling, facilitating stem/progenitor cell population maintenance [60]. Pericytes comprising the vasa vasorum in the tunica adventitia also contribute to the stabilization of tunica adventitia microvessels [37]. Pericytes in these locations have been reported to differentiate into SMC lineages, a characteristic which could allow for their involvement in microvessel formation within the aortic wall, either through direct recruitment or secretion of angiogenic growth factors [1]. Based on this characteristic, they could thus play a role in the maintenance of aortic wall homeostasis, remodeling and repair. Finally, via the vasa vasorum, they can also influence the behavior and function of the tunica media, as the vasa vasorum of the thoracic aorta extend all the way through to the outer layers of the tunica media [1]. Under pathologic conditions, pericytes in the tunica adventitia contribute to angiogenesis during vascular remodeling [61] and atherosclerosis [3], as well as influence aneurysm development in the ascending thoracic aorta [7,8].

Pericytes also contribute to the development of the aorta, as observed in animal models. During embryonic development, the formation of the aortic wall in the early stages of chick development comprises the recruitment of transient vascular SMC populations (primary populations) from the splanchnic mesoderm, for derivation of the aortic floor. This event is later followed by the recruitment of additional vascular SMC populations (definitive populations) from the sclerotome of the adjacently developing somite [62]. Mural cells, including vascular SMCs and pericytes, can exhibit differential embryological origins depending on location [63], a phenomenon often described as the ‘multi–site hypothesis for the ontogeny of mural cells’ [64]. In general, in the area of the forebrain, face, neck and truncus arteriosus, vascular SMCs derive from the cephalic neural crest; in the area of the heart septum and proximal cardiac artery, vascular SMCs also derive from the cephalic neural crest; in coronary veins, the vascular SMCs of the developing vascular walls derive from the myocardium whilst in coronary arteries these originate from the epicardium; and, finally, in the area of trunk and aortic wall, vascular SMCs, along with aortic pericytes, all originate from adjacent developing somites, and, in particular, the sclerotome. In these same studies, pericytes have also been observed in the basal lamina of the chick aorta. These pericytes are identified on the basis of their localization around assembling ECs with which they share a basal membrane; they are also identified by the expression of α–SMA, fibronectin and N-cadherin, as well as the presence of intercellular contacts, such as tight and gap junctions [63]. Recruitment of mural cells, such as vascular SMCs and pericytes, to developing blood vessels, is aided by platelet–derived growth factor BB (PDGF–BB)/PDGFRβ signaling. Further recruitment, differentiation and stabilization of mural cells is supported by TGFβ1, Notch and Epidermal growth factor (EGF) signaling as well [64], at least based on studies in developing zebrafish. Mural cells thus completely envelop the dorsal aorta during zebrafish embryonic development. In in vivo experiments where mural cell function is prevented, via the inclusion of a dominant negative form of the PDGFRβ gene that is heat–shock inducible, or, alternatively, via the use of zebrafish deficient in vascular SMC function due to the absence of PDGFRβ, the dorsal aorta diameter is larger. Similar effects are observed in vitro, with joint cultures of ECs and pericytes in the 3D collagen matrix assembling into vascular tubes that possess more restricted diameters, as opposed to seeding of ECs alone. In the latter cases, the vascular tube diameter continues to increase with time [64]. Thus, mural cells (vascular SMCs and pericytes) generally contribute to the stabilization of the dorsal aorta diameter during early development. This occurs via secretion of basement membrane components, which in turn prevent excessive elasticity of the developing dorsal aorta wall and thus restrict excessive expansion [64]. Both vascular SMCs and pericytes could represent phenotypic variations in the same cellular lineage in these studies [65,66].

### 4.3. Tunica Intima and Cell Populations

The tunica intima, as already stated, is the innermost layer of the aortic wall, abutted from the tunica media via the IEL [21,22]. The tunica intima is generally composed of a single layer of squamous ECs which rest upon a basement membrane and are in direct contact with the vascular lumen. The latter comprises ECM proteins such as collagen type IV; this is the principal component of the basement membrane, along with collagen type XVIII. In addition, various laminin isoform proteins (namely α4 and α5) and heparan sulfate proteoglycans, such as perlecan, can be identified as well [67]. The basement membrane usually supports the endothelial layer [68,69]. In total, the tunica intima along with the tunica adventitia together comprise approximately 1/3 of the aortic wall. Thus, the tunica media is the largest component of the aortic wall, save for regional variations, as based on morphometric studies of the porcine aorta [70]. Furthermore, in contrast to the tunica media and tunica adventitia, the tunica intima remains uniform in thickness throughout the length of the aorta [71]. The subendothelial layer lies below the basal lamina and comprises a loose connective tissue matrix composed mainly of collagen type III and type IV, elastin and proteoglycans such as perlecan, as well as thrombomodulin [72]. In the aorta, this layer may be thicker than most vessels and can contain various cell populations as well [73].

Cells found in the subendothelial layer include fibroblasts, as well as myointimal cells resembling SMCs [73]. SMCs can also be found here; however, these do not appear to organize in layers, as occurs in the tunica media. These SMCs are instead dispersed within the ground matrix. SMC populations in the subendothelial layer are identified by expression of α–SMA, though cells morphologically resembling vascular SMCs stain negative for α–SMA. Additional cellular types include immune cell populations, including lymphocytes that may happen to infiltrate this layer from the luminal endothelial layer, as well as macrophages and phagocytes, expressing both CD68 and α–SMA. Hematopoietic cell populations generally represent approximately ~5% of the total cells present in the subendothelial layer [3]. Stem/progenitor cells have been identified in this location as well, comprising the so-called subendothelial zone. Hematopoietic stem/progenitor cell populations can be identified as CD133+/CD45+, mesenchymal stem cell populations as CD44+/CD73+/CD90+/CD34–/VEGFR2– and, finally, endothelial precursor populations as CD34+/VEGFR2+/CD31– [3].

**Figure 2:**
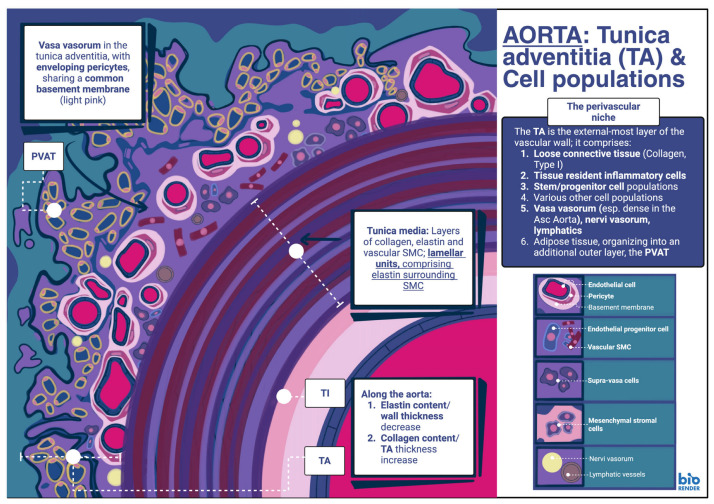
Cells and progenitor populations of the tunica adventitia. Picture of the aorta, at the level of the ascending thoracic segment, detailing aspects of the wall including the adventitia and vasa vasorum. The microvessels present in the tunica adventitia and the perivascular adipose tissue (PVAT) are also shown with the adjacent pericytes surrounding each microvessel. The endothelium is depicted with background coloration in both the main lumen as well as the microvessel lumen. Additional components of the adventitia are included as well, including nerves, lymphatics and various progenitor populations in the perivascular niche. Asc Aorta, ascending aorta; PVAT, perivascular adipose tissue; TA, tunica adventitia; TI, tunica intima; SMC, smooth muscle cell. [Illustration created digitally with the painting application ‘Procreate^®^’ composite graphic assembled with BioRender.com. (Created in BioRender. Stougiannou, T. (2026) https://BioRender.com/t04isqk, accessed on 16 March 2026)].

**Figure 3:**
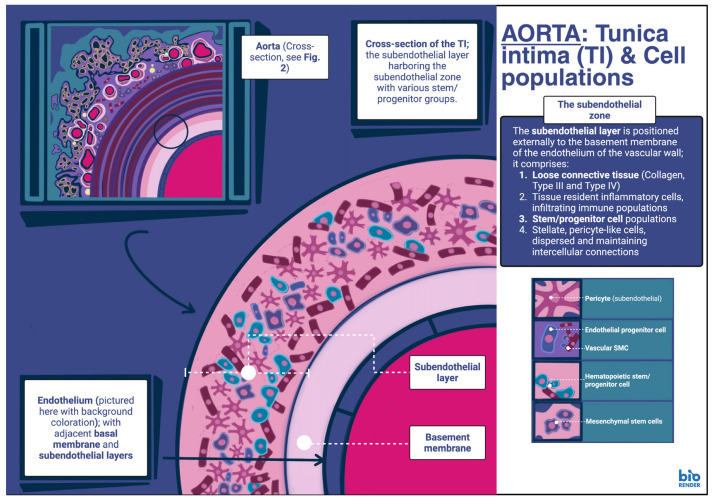
Cells and progenitor populations of the tunica intima. Picture of the aorta, at the level of the ascending thoracic segment, detailing aspects of the wall including the endothelium and tunica intima. TI, tunica intima. [Illustration created digitally with the painting application ‘Procreate^®^’ composite graphic assembled with BioRender.com. (Created in BioRender. Stougiannou, T. (2026) https://BioRender.com/g0zprto, accessed on 16 March 2026)].

Cellular populations expressing proteins characteristic of microvascular pericytes have been identified in the subendothelial layer of the intima as well; some of these proteins include antigen 3G5, an O–sialoganglioside normally expressed on the surface of microvessel pericytes. Cells expressing this protein have been observed to have stellate morphology in early studies, forming a cellular network in the subendothelial layer of large arteries. Similar cells are also identified in such studies in the tunica adventitia, enveloped by ECs [74], and have been termed pericyte–like cells [3]. They have been identified in both the physiologic and atherosclerosis-laden tunica intima of the aorta in both bovine and human specimens [2]. While the expression of 3G5 is associated with quiescent pericytes, expression of another protein, 2A7, also identified in these pericyte–like cells, signifies actively proliferating pericyte–like cells, perhaps in the process of active angiogenesis. Furthermore, in vitro studies show an increase in the fraction of 2A7 cells, along with a reduction in the 3G5 fractions, upon stimulation with modified low–density lipoprotein (LDL). This reduction in 3G5 cells is associated with increased intracellular deposition of LDL as well. Thus, lipid accumulation in association with atheroma formation usually accompanies changes in pericyte–like cell populations in the subendothelial layer of the aortic intima [75]. Similarly, pericyte–like cells expressing α–SMA/2A7/3G5/CD68, are identified in the aortic intima as well, though these may represent specialized pericyte populations with phagocytic activity [3,76,77]. These stellate cells indeed form a continuous network in the proteoglycan–rich subendothelial layer of the aortic intima [78]. Often, this network dissociates in pathological states, such as atherosclerosis [79], with intracellular accumulation of lipids identified as one of the reasons for this dissociation [3]. In addition, during atherosclerosis and the local inflammatory microenvironment that occurs as a result, pericytes in the intima can be induced to proliferate and migrate, causing localized angiogenesis. Alternatively, they can be induced to transdifferentiate into other cell types as well, owing to their multipotent differentiation capabilities, further contributing to and propagating vascular wall remodeling [3,80] (Figure 2 and Figure 3, Table 1).

## 5. Thoracic Aortic Aneurysm (TAA) and Pericytes

### 5.1. Thoracic Aortic Aneurysm (TAA) and the Tunica Adventitia

Thoracic aortic disease encompasses disease states that affect the aorta, including thoracic aortic aneurysm (TAA) and thoracic aortic dissection (TAD) [138]. An aneurysm can be generally described as a localized or more diffuse dilatation of the vascular wall, usually reaching a diameter at least 1.5 times the normal vascular diameter. Aneurysms can be classified according to location; in the case of TAA, these describe aneurysms of the thoracic aorta. Usually, ~60% of TAA affect the aortic root and ascending aorta, and ~40% affect the descending thoracic aorta [34]. Aneurysm pathology affecting the thoracic aorta can be mainly attributed to cystic media degeneration [36], though this phenomenon has been more recently described as ‘mucoid extracellular matrix accumulation’ or MEMA. MEMA, often a phenomenon that occurs naturally during the physiological aging process, can be accelerated by pathophysiological processes such as hypertension, presence of bicuspid aortic valve or various genetic conditions. Finally, additional etiological conditions that can lead to TAA in the aortic root and ascending thoracic aorta include genetic syndromes (Marfan syndrome, vascular Ehlers–Danlos), as well as non-syndromic factors such as bicuspid aortic valve [34]. In the descending thoracic aorta, on the other hand, etiological factors differ and can include atherosclerotic vascular wall pathologies, and infectious and inflammatory conditions [34].

Often, TAA in the aortic root and ascending thoracic aorta can continuously progress, reaching an unstable state that causes a tear in the tunica intima, leading to acute aortic dissection (TAD). During this state, blood flows between the vascular wall layers. Type A dissections, per the Stanford classification, usually involve the ascending thoracic aorta and may extend well into the descending thoracic aorta as well as the abdominal aorta, while Type B dissections usually originate from the descending thoracic aorta, after the subclavian artery branch point [138]. While the prevalence of TAA compared to aneurysms in the abdominal aortic segment is comparably lower, the prognosis is often more serious with higher mortality [71]. Surgery usually follows presentation, with a high reported early post-operative mortality, often at approximately ~15%. Furthermore, apart from early mortality in the post-operative period, other complications can include bleeding and CNS dysfunction. The latter can often be attributed to global cerebral hypoperfusion during the procedure as well as embolism, while risk factors for this occurrence usually include advanced age and lesions/procedures in the aortic arch, as well as aortic arch clamping [34].

The ECM of the aortic adventitia has a specific composition, which has already been detailed in Section 3: Aorta; however, specific pathologies often alter the composition of the aortic adventitia. Examples include the accumulation of collagen and elastin, leading to increased stiffness in the general arterial and pulmonary arterial hypertension [7]. Additionally, a decrease in collagen and elastin content in the adventitia can occur with aging, which is also thought to contribute to the development of aortic aneurysm [7]. In such cases, the pendulum of physiological ECM remodeling, normally maintained by a balance between new ECM synthesis and degradation, is shifted towards degradation. These alterations mirror similar changes occurring in the media, as the pathology generally affects all three layers of the aortic vascular wall [71]. Collagen, including collagen type I and type III, is synthesized by fibroblasts in the tunica adventitia, among others [139]. During disease states and, more specifically, in patients with TAA and TAD, collagen production by adventitial fibroblasts is compromised as well. In turn, this leads to weakening in the adventitia layer, further promoting the outward expansion of the wall [140]. Finally, soluble components found within the adventitia can also be altered and contribute to the pathogenesis. For example, in the adventitia of TAA specimens, higher levels of the anti-angiogenic factor thrombospondin–1 (TSP–1) have been identified, along with reduced levels of pro-angiogenic factors such as vascular endothelial growth factor (VEGF), angiopoietin 2 and fibroblast growth factor 2 (FGF2 or basic fibroblast growth factor [bFGF]) [7,141].

Various studies report a reduction in pro-angiogenic growth factors in the tunica adventitia of TAA specimens [36,142], in turn leading to vasa vasorum disruption. This will eventually lead to a reduction in vasa vasorum-mediated blood flow in the tunica media. As a result, localized tissue hypoxia in the tunica media ensues. Chronic hypoxia in the tunica media is often associated with the upregulation of genes such as GLUT1 in this location, induced by hypoxia–inducible factor–1 alpha (HIF–1α) and denoting a state of anaerobic glycolysis [36]. This however, may also be one of the causes of the reduction in angiogenic growth factors in the tunica adventitia, such as HIF–1α and its various downstream targets (VEGF and metallothionein), along with the general reduction in angiogenic signaling processes [36,142]. Disruptive changes, as a result of disruption in available angiogenic growth factors, can include a reduction in vasa vasorum density [8,36], though this has only been observed in tricuspid aortic valve specimens in some studies [36]. Conversely, in patients with bicuspid aortic valve, as well as during physiological aging, no correlation between vasa vasorum density and aortic diameter exists. This reduction in vasa vasorum density can also oftentimes be accompanied by an increase in the vasa vasorum lumen [8,36]. When measured collectively, total luminal area increases in both tricuspid and bicuspid aortic valve specimens. At the same time, a reduction in the density of small–sized vasa vasorum and increase in the density of larger-sized vasa vasorum occur, events which positively correlate with ascending thoracic aortic diameter as well [36]. Finally, vasa vasorum microvessels can also undergo remodeling, evidenced by increased wall thickness. Once again, however, this only occurs in aortic specimens from patients with tricuspid aortic valves [36].

Localized tissue hypoxia usually stimulates cognate signaling pathways, including those that comprise activation of HIF–1α and upregulation of downstream gene targets, such as *metallotheionin–1 alpha* (*Mt–1α*) and *Vegfa*, in target tissues. During chronic tissue hypoxia, however, there can be downregulation of VEGF signaling instead. As a result, downregulation of the corresponding angiogenic processes in ECs occurs, through a mechanism involving HIF–1α [143]. VEGF-mediated angiogenic processes are further downregulated due to the reduction in Mt–1α in the tunica adventitia. Thus, overall, chronic hypoxia in the tunica media may lead to downregulation of HIF–1α and VEGF signaling in the tunica adventitia, a theory corroborated by the observed disturbances of vasa vasorum in the tunica adventitia. This sequence of events, eventually leading to TAA, may be initially stimulated due to local alterations in the vasa vasorum network of the tunica adventitia. In addition, inflammatory cell infiltration in the tunica adventitia can also disrupt vasa vasorum. These changes further contribute to pathological changes in the tunica media of the aorta in cases of TAA as well as TAD. This occurs as, in the latter, the dissection usually propagates along the vasa vasorum path, at the boundary between the tunica media and the tunica adventitia. Throughout these above-described processes, there are some differences in the pathophysiological features of the tunica adventitia between tricuspid and bicuspid aortic valve specimens. For example, GLUT1 upregulation only occurs in tricuspid aortic valve specimens, along with a greater reduction in vasa vasorum density [36].

Due to the increased rate of angiogenesis in the tunica media as a result of this chronic hypoxia, as well as the vasa vasorum remodeling that occurs, the vasa vasorum penetrating the tunica media are structurally immature. These new vessels are oftentimes characterized by the absence of pericyte coverage, disruption of cell–to–cell contacts and incomplete or absent basal lamina. This eventually facilitates the accumulation of plasma proteins in the tunica media [144]. This observation is corroborated by the concurrent increase in angiogenic growth factors that promote microvessel formation (angiopoietin 1, angiopoietin 2, VEGF, and FGF) in the tunica media of TAA specimens, coupled with the increase in levels of factors that prevent angiogenesis (TSP–1). In general, these new structurally immature microvessels are observed near areas of mucoid degeneration and ECM degradation in the tunica media, likely allowing for invasion by new microvessels in these locations [144]. Unlike other studies [36], however, this observed angiogenesis in the tunica media has not been attributed to hypoxia in this study [144].

### 5.2. Thoracic Aortic Aneurysm (TAA) and Pericytes

In the tunica adventitia of the thoracic aortic wall, FGF2 (also known as bFGF) is normally synthesized and secreted by multiple cell types, including adventitial fibroblasts. These cells represent a phenotypically heterogenous population and generate, among others, components of the ECM, including collagen type I and type III. Though normally quiescent, adventitial fibroblasts can be stimulated to produce various growth factors and proteins that stimulate processes such as inflammation and angiogenesis [139]. FGF in tissues is usually bound to components of the ECM, such as heparan sulfate proteoglycans [145], and can be released during biological processes that result in degradation of the ECM [139]. FGF synthesis by fibroblasts can also be enhanced by factors such as transforming growth factor beta (TGF–β) and tumor necrosis factor alpha (TNF–α) [146]. FGF is also produced and secreted by pericytes, contributing to EC proliferation and angiogenesis in the tunica adventitia [1,7,102]. In this manner, pericytes contribute to vascular remodeling within the tunica adventitia of the aorta, maintaining homeostasis of the vascular wall [1]. FGF2 signaling can also be used to direct differentiation of pluripotent stem cell groups towards the pericyte phenotype as well as maintain plasticity of pericyte populations and contribute to microvessel pericyte coverage. Maintenance of the pericyte phenotype usually occurs via effects on phosphoinositide 3 kinase (PI3K)–protein kinase B (AKT) signaling [7].

As with other growth factors that promote angiogenesis [36,142], FGF2 levels are reduced in the tunica adventitia of TAA specimens, a difference observed between physiological and pathological groups. Usually, no differences in expression across different age and sex groups is observed [7]. However, some studies do report a reduction in FGF2 levels with increasing age [147]. As a result of this effect on FGF2 levels, and based on further study of single–cell ribonucleic acid (RNA) sequencing data, pericytes in the tunica adventitia of TAA specimens have been shown to display a change in phenotype [7]. Namely, processes related to muscle tissue, such as muscle structure, development, contraction and differentiation, are enriched in these cells compared to wild-type groups. Examples include the upregulation of myosin heavy chain 11 (MYH11), transgelin (TAGLN or smooth muscle protein 22 alpha [SM22]), and myosin light chain 9 (MYL9). Other genes related to muscle and contractile function, however, such as calponin 1 (CNN1) and actin alpha 2 smooth muscle (ACTA2), show no difference in expression. Loss of FGF2 in the ECM of aneurysmal tunica adventitia will then lead to inhibition of Mitogen–associated protein kinase (MAPK) or PI3K–AKT signaling in target pericytes. This eventually stimulates the phenotypic switch towards the contractile phenotype. At the same time, the generation of growth factors contributing to angiogenesis, such as VEGF, is downregulated as well [7]. The pericytes in the study associated with these results are negative for CD31 expression and are further identified via expression of CD146 (CD31–/CD146+ for magnetic bead separation); they also express NG2 (also known as CSPG4) and PDGFRβ [7].

Affected pericytes exhibit enhanced contractility and reduced ability for migration, as well as reduced capability for supporting angiogenesis; changes in cellular behavior can be further observed through collagen compaction assays in vitro [7]. This change in behavior may have deleterious effects on the function of the vasa vasorum in the tunica adventitia. For example, contractile pericytes could affect blood flow through the local vasa vasorum [7], contributing to the local hypoxia observed in other similar experiments [36]. This can then, in turn, trigger additional downstream processes, eventually contributing to the development of TAA or TAD [7,148,149]. Furthermore, this could also influence and contribute to the tunica media hypoxia identified in other similar studies [36]. However, additional experiments bridging FGF2 expression, evaluating blood flow in tunica adventitia microvessels, and the resulting, if any, hypoxia in the tunica media may need to be carried out to further evaluate this hypothesis. This is particularly suitable, since relevant groups in the adventitia can include pericytes identified by the biomarker expression profile of CD34–/CD31–/CD146+ and adventitial cells (or supra–vasa cells) identified as CD34+/CD31– [1], along with intermediate populations characterized as CD34+/CD146+ [150]. Careful observation of the behavior in all these cell populations in these conditions could thus yield a breadth of relevant information. In addition, CD34+ adventitial cells are often regarded as a pericyte progenitor population [1] and thus care must be given to appropriately identify and track these pericyte–like populations in any planned study. In this manner, a clearer view of the contributions of each population to functions related to adventitia blood flow via vasa vasorum networks may be gleamed.

As already stated, the loss of FGF2 affects PI3K–AKT and MAPK signaling in CD31–/CD146+ pericytes [7]. FGF2 can normally stimulate PI3K–AKT and MAPK signaling, triggering multiple biological processes, including signaling cascades involved in angiogenesis, via binding to immunoglobulin–like FGF receptors (FGFR) [145]. Upon receptor dimerization and autophosphorylation, PI3K catalyzes conversion of PIP2 to PIP3, which in turn allows for recruitment and activation of AKT. Conversely, PTEN, a phosphatase enzyme, catalyzes the PIP3 to PIP2 transition, preventing constitutive activation of this pathway. Eventually, angiogenesis is triggered via various mediators, including HIF–1α, VEGF and others [151]. With MAPK, stimulation by FGF2 leads to downstream activation of the protein kinases Mitogen–associated extracellular signal–regulated kinase (MEK) and extracellular signal–regulated kinase (ERK) [142,152]. As FGF2 signaling normally maintains the CD31–/CD146+ pericyte phenotype in the vasa vasorum of the thoracic aorta, the removal or reduction in FGF2 in the local environment inhibits MAPK and PI3K–AKT signaling in these cells. This eventually leads to upregulation of *CNN1* and *SM22*, along with increased levels of CNN1, SM22 and αSMA proteins. These effects can be recreated in vitro using separate inhibition of both MAPK via the ERK inhibitor PD98059 and PI3K–AKT via the inhibitor LY294002 [7]. In addition, both MAPK and PI3K–AKT signaling pathways inhibit migratory capacity in these pericyte populations [7]. In general, no effect on native pericyte markers is observed, except from an increase in *PDGFRβ* expression seen with MAPK signaling inhibition [7]. In turn, this facilitates appropriate identification of the affected populations, even after the observed change in gene expression.

Other similar studies examining adventitia pericytes in the context of TAA development and progression reveal hyperactivation of MEK/ERK signaling as an etiological mechanism behind the pathological changes occurring in the vasa vasorum in such cases [8]. This is evident by the increase in phosphorylated ERK in the examined pericyte populations, along with the reduced capacity for migration. The pericyte populations examined in this study normally display a contractile phenotype with increased capacity for migration [8], as well as a capability for supporting angiogenesis [8]. The latter effect is normally seen in physiological pericyte populations evaluated in other similar studies as well [7]. These characteristics can be restored in pericytes isolated from affected tissues after the use of the MEK inhibitor PD0325901. Affected pericytes also express increased levels of pro–inflammatory cytokines, including interleukin–6 (IL–6) and monocyte chemoattractant protein–1 (MCP–1) [8]. These factors could further aggravate aortic wall inflammation, more prominent in abdominal aortic lesions, which contributes to aortic aneurysm pathophysiology, as recreated in in vivo experimental models, including the hyperlipidemic, angiotensin II infusion and intraluminal elastase models [71]. In addition, this provides further evidence for the contribution of inflammatory processes in the development of TAA [8,34,153] originating in the adventitia [8].

It is the effects on MAPK and PI3K–AKT signaling in pericytes that also affect their abilities to contribute to and support angiogenesis. For example, restoring FGF2 signaling in CD31–/CD146+ pericyte populations with a pathologically contractile phenotype in turn restores VEGF expression [7]. VEGF expression in vitro is prevented by PI3K–AKT inhibition, via the inhibitor LY294002. On the other hand, the MAPK signaling pathway does not seem to be involved in the regulation of VEGF expression in tunica adventitia pericytes identified by the biomarker expression profile CD31–/CD146+. This is most likely due to the observation that MAPK inhibition via the ERK inhibitor PD98059 does not lead to considerable changes in VEGF levels in these populations [7]. In these same pericytes, exposure to FGF2 also reduces levels of growth factors that prevent angiogenesis, such as TSP1; TSP1 can be upregulated in vitro with MAPK inhibition via the ERK inhibitor PD98059. On the other hand, PI3K–AKT inhibition via the inhibitor LY294002 has no effect on TSP1 expression [7].

Conversely, in similar studies of tunica adventitia pericytes, the inhibition of MEK signaling via the inhibitor PD0325901 restores pericytes’ capability for supporting angiogenesis, as MEK/ERK upregulation prevents physiologic pericyte function in these populations [8]. These cell group populations also demonstrate increased expression of growth factors that prevent angiogenesis, such as TSP–1 and angiopoietin 2; once again, the inhibition of MEK signaling via the inhibitor PD0325901 restores normal levels of angiopoietin 2 expression [8]. Compared to the CD31–/CD146+ pericyte populations evaluated in similar experiments [7], these pericyte groups exhibit differences in cellular attributes, as contractility is reported to be a physiological characteristic in the pericyte populations examined [8]. This difference could be attributed to differential baseline characteristics in the pericyte populations examined in each study or, alternatively, it could point to a need for tight regulation of MEK/ERK (MAPK) signaling or the interaction between MEK/ERK (MAPK) and PI3K–AKT signaling for the maintenance of physiological pericyte expression/function in the vasa vasorum of the tunica adventitia [7,8]. The identification of pericyte populations is often carried out using CD146, along with other associated markers detailed in previous sections [1]; however, CD146 as a marker is non–specific [102], with many groups describing CD146– pericyte populations expressing, among others, CD34 [154,155]. However, CD34 expression has been attributed to adventitia, or supra–vasa cells, by other research groups, at least in studies of pericytes identified in the tunica adventitia of the thoracic aorta [1]. This could point to differences in marker expression amongst pericytes found in different locations across the cardiovascular system, or, alternatively, different but overlapping populations with similar characteristics.

FGF has been either supplemented or modified in various studies examining novel methods for aortic aneurysm repair. TAA models in canine specimens have been used to evaluate the effects of supplemented FGF with other therapeutic approaches. Some of these approaches comprise a process of direct cell transplantation composed of skeletal myoblasts/fibroblasts and collagen gel along with bFGF [156]. Others include the use of biodegradable polyglycolic acid (PGA)–derived wrapping material impregnated with bFGF, for implantation in the descending thoracic aorta [157]. In either case, provision of exogenous FGF either facilitates the favorable effects of the cell therapy [156], or leads to increased angiogenesis in the tunica adventitia. The latter is evident by a thickened adventitia, rich in vascular networks after application in canine models [157]. The application of PGA–derived implants infused with bFGF in canine models of thoracic aortic dissection has also been carried out; the use of the biodegradable implants preserves vascular compliance after TAD repair, owing to the preservation of elastic fibers in the vascular wall. In this case, however, contrary to what would be expected, vascularity in the aortic wall is reduced after implantation. This could be attributed to the timing of histology evaluation in this model; examination is carried out at time of euthanasia only and serial examinations throughout the post-operative period are absent. Alternatively, the dose of bFGF administered in this experiment may not be ideal for the full angiogenic effect to take place [158].

Newer studies involve use of nuclear antisense transcripts (NATs) derived from the strand complementary to the coding strand of a target gene; these transcripts generally exert a suppressive function on the target gene [159]. In the case of unstable atherosclerotic plaques and abdominal aortic aneurysm (AAA), the long non-coding RNA (lncRNA) Nucleoside diphosphate–linked moiety X motif 6 (NUDT6), a NAT specific for the FGF2 gene, is often increased, causing FGF2 downregulation. Normally, NUDT6 can be upregulated by factors such as oxLDL. The inhibition of NUDT6, and, as a result, the upregulation of FGF2, has a favorable effect on disease progression and severity, reducing the rate of aneurysm growth and risk of atherosclerotic plaque rupture. In this case, however, the effect is mainly mediated via FGF2 functions on vascular SMC proliferation and apoptosis [160]. Other studies evaluate the role of FGF signaling and its inhibition in ECs of the tunica intima. Inhibition of the FGF/FGFR signaling cascade via administration of platelet factor 4 (PF4), inhibits migration and angiogenic functions in these populations. This, in turn, prevents propagation of TAA and TAD [161]. As opposed to previous examples, FGF function and angiogenesis in the target population need to be prevented here for a favorable outcome to occur. Thus, knowledge not only of the target molecule, but also of the physiologic process, as well as the target cellular population that needs to be addressed, is required. The observation that FGF signaling can result in differential outcomes, depending on the area and target cell population within the vascular wall, may also necessitate targeted delivery of FGF supplementation or FGF modifying therapies for the prevention of TAA and TAD in future studies.

## 6. Conclusions

Pericytes are stellate-like cells identified in microvascular beds across multiple organ systems, often seen enveloping these microvessels, contributing to vascular stability and facilitating or supporting angiogenesis. The aorta, as with larger vessels, contains microvessels in the outer layers, or tunica adventitia, which contribute to maintenance and homeostasis of the aortic wall. As expected, dysfunction or dysregulation in the tunica adventitia microvessels, or vasa vasorum, contributes to many different pathophysiological processes, including TAA or TAD. Pericytes, as an integral component of these microvessels, can contribute to this dysfunction; recent studies point to FGF2 as well as MEK/ERK (MAPK) and PI3K–AKT signaling dysregulation as contributing factors, though some results seem conflicting. It is without a doubt that through additional research in this research niche, the contribution of tunica adventitia pericytes in TAA development will be further illuminated, facilitating the identification of signaling pathways and physiological processes interconnecting these variables.

## Figures and Tables

**Table 1:** Pericytes and marker expression. Overview of biomarkers used to differentiate between the main cellular groups found in the vascular wall (pericytes, endothelial cells [ECs], vascular smooth muscle cells [SMCs]). The function of each marker, in relation to relevant processes associated with angiogenesis and vascular function, if present, is included as well. ACTA2, actin alpha 2, smooth muscle; AKT, protein kinase B (PKB); ALP, alkaline phosphatase; AMP, Adenosine monophosphate; ASC, adipose stem cell; BCR, B–cell receptor; CD11b/14/19/31/34/44/45/56/68/73/79a/90/105/117/146/201, CD11b/14/19/31/34/44/45/56/68/73/79a/90/105/117/146/201 molecule; CM, cardiomyocyte; CNN, calponin; CNS, central nervous system; CPC, cardiac progenitor cell; CSPG4, chondroitin sulphate proteoglycan 4; E8.5/9, mouse embryonic day 8.5/9 of development; ECM, extracellular matrix; EPC, endothelial progenitor cell; EPCR, endothelial protein C receptor; ERK1/2, extracellular signal–regulated kinase 1/2; FAK, focal adhesion kinase, protein tyrosine kinase 2 (PTK2); FGF/1, fibroblast growth factor/1; GATA4, GATA–binding protein 4; GF, growth factor; GM1, Monosialotetrahexosylganglioside; GM2, Monosialotriahexosylganglioside; GPI, Glycosylphosphatidylinositol; HLA–DR, human leukocyte antigen, D–related (DR); HLA–DRA/B1/B3/B4/B5, human leukocyte antigen, D–related (DR); HMW–MAA, high molecular weight melanoma–associated antigen; HSP27, heat–shock protein 27; HSPC, hematopoietic stem/progenitor cell; HUVEC, human umbilical vein endothelial cell; ICAM1, intercellular cell adhesion molecule 1; IF, intermediate filament; IFN–γ, interferon gamma; IL–17, interleukin–17; IVC, inferior vena cava; Ig–α, immunoglobulin alpha; IgM, immunoglobulin M; LAMP, lysosomal–associated membrane protein; LDL, low–density lipoprotein; LPS, lipopolysaccharide; Ly6A/E, lymphocyte antigen 6A/E; MCAM, melanoma cell adhesion molecule; MCP1, monocyte chemoattractant protein 1, C–C Motif chemokine ligand 2 (CCL2); MCSP, melanoma chondroitin sulphate proteoglycan; MD2, myeloid differentiation protein 2; MHC II, major histocompatibility complex II; MSC, mesenchymal stromal cell; Mef2, myocyte enhance factor 2; Mg, magnesium; NF–κB, Nuclear Factor Kappa B; NG2, neural glial antigen 2; NK cell, natural killer cell; OPC, oligodendrocyte precursor cell; PDGF–A/B/C/D/BB, platelet–derived growth factor A/B/C/D/BB; PDGFRβ, platelet growth factor receptor beta; PI3K, phosphoinositide 3 kinase; PLT, platelet; PROCR, protein C receptor (gene); PVAT, perivascular adipose tissue; RBC, red blood cell; SCF, stem cell factor; SM–MHC, smooth muscle, myosin heavy chain, or myosin heavy chain 11 (MYH11); SMC, smooth muscle cell; SCA–1, stem cell antigen–1; Src, Src proto–oncogene, non–receptor tyrosine kinase; TA, tunica adventitia; TCR, T–cell receptor; TAGLN, transgelin; TEK, TEK receptor tyrosine kinase; TGFβ/1, transforming growth factor beta/1; TI, tunica intima; TLR4, Toll-like receptor 4; TNAP, tissue non–specific alkaline phosphatase; Tie2, TEK receptor tyrosine kinase (TEK); UPAR, urokinase plasminogen activator receptor; VEGFA/C/D/121/164, vascular endothelial growth factor A/C/D/121/164; VV, vasa vasorum; VWF, von Willebrand factor; WT1, Wilm’s tumor protein; Zn, zinc; bEGF, basic fibroblast growth factor; c–Kit, KIT proto–oncogene, receptor tyrosine kinase; mAb, monoclonal antibody; mTOR, mammalian (or mechanistic) target of Rapamycin; oxLDL, oxidized low–density lipoprotein; sCD14, soluble CD14 molecule; α–SMA, actin alpha 2, smooth muscle (ACTA2).

Marker	Gene Name	Pericyte Marker	Cardiovascular/ Aortic Wall	Information	References
2A7 (NG2)	*MCSP*, (also known as *CSPG4*)	YES	NG2 is also found in vascular SMCs (large vessels including aorta), CM	Antigen 2A7 is expressed in activated pericytes; it has since been identified as MCSP (or Chondroitin sulfate proteoglycan or NG2). The term ‘antigen 2A7’ appears in early studies of stellate, pericyte–like cells in the subendothelial layer of large vessel intima and the effects of atherosclerosis on their morphology. Both 2A7 and 3G5 are named after the mAb used to identify an antigenic target, with mAb 2A7 identifying HMW–MAA (also known as CSPG4 or MCSP) and mAb identifying an O–sialoganglioside compound.	[2,74,75,76,78,79,80,81,82,83]
3G5	O–monosialoganglioside	YES	N/A	Identified on the surface of stellate, pericyte–like cells in the subendothelial layer of the aortic intima (assembled into a network in this layer). Antigen 3G5 is specific for microvascular pericytes and is often present in cells characterized by membrane processes. Both 2A7 and 3G5 are named after the mAb used to identify an antigenic target; mAb 2A7 identifies HMW–MAA (also known as CSPG4, or MCSP); mAb identifies an O–monosialoganglioside compound, possessing intermediate mobility between gangliosides GM1 and GM2.	[3,75,80,81,82,83]
Alkaline phosphatase	*ALP* *TNAP*	YES	Aortic valve interstitial cells (pathologic calcification)	Alkaline phosphatases are isoenzymes expressed on the cell membrane surface, along with co-factors (Zn, Mg), they catalyze hydrolysis of organic phosphate esters in the ECM. TNAP expressed mainly in liver and bone, corresponds to circulating fraction of alkaline phosphatase; overexpression associated with aortic calcification (osteochondrogenic transformation in aortic pericytes). Expressed by progenitors which give rise to fetal myoblasts during embryonic development, in turn generating skeletal and SMC populations, as well as pericytes; TNAP+ pericytes will eventually give rise to skeletal muscle and SMC populations.	[4,84,85]
Angiopoietin 1	*ANGPT1*	YES	Vascular SMC, fibroblasts	Soluble vascular growth factor, involved in angiogenesis and vascular stabilization/maturation; it binds the Tie2 receptor, mediating interactions that contribute to vessel stabilization. Angiopoietin 1 is expressed by pericytes and many other cell types; in the context of pericyte–EC interactions, angiopoietin 1 binds Tie2 on the surface of ECs, is involved in EC quiescence/survival and vascular maturation via AKT–mediated regulation of FOXO1.	[20,86]
c–Kit (CD117)	*KIT*	NO	EC	Transmembrane protein receptor, tyrosine kinase family. Expressed on the surface of many different cells, including EC. Binds SCF, and regulates cell proliferation and cell–to–cell adhesion, which are distinguishing features of hemogenic ECs, compared to non-hemogenic EC populations. Stimulation of c–Kit/SCF signaling in EC activates pro-angiogenic reactions associated with vascular tube assembly, stimulates proliferation/angiogenesis processes in HUVEC via AKT–mTOR and ERK signaling; expression of c–Kit in large vessel EC (IVC) correlates with degree of intimal thickness, intimal stenosis in association with renal failure.	[87,88,89]
CD31	*PECAM1*	NO	EC	Expressed by ECs in cell–to–cell junctions, involved in adhesion between immune cells and ECs, regulates leukocyte transmigration across the endothelial surface during inflammation, and is involved in angiogenesis.	[1,90]
CD34	*CD34*	YES	HSPC	Hematopoietic progenitor cell antigen, functions in cell adhesion, anchoring HSPC to surrounding matrix in bone marrow. Involved in lymphocyte–EC (lymphoid tissues) interaction, also interacts with L– and E–Selectin. Involved in maintenance of ‘stemness’ and regulation of cell proliferation in undifferentiated stem/progenitor cell populations; involved in regulation of cell migration during wound healing and angiogenesis. Expressed by a subset of pericytes (α–SMA+ CD31–) in the tunica adventitia; CD34+ pericytes represent ~15% of non–endothelial groups (CD31–) in the perivascular niche of the ascending thoracic aorta, compared to ~32% in the perivascular niche of adipose tissue. Absence of CD34 is one of the defining characteristics of mesenchymal stem cell populations, though this requirement is not absolute.	[1,91,92,93]
CD44	*CD44*	NO	EC (CD44V8–10/CD44E)	Cell surface glycoprotein and adhesion molecule involved in cell–to–cell and cell-to-ECM interactions; facilitates cell trafficking/homing towards lymph nodes, participation in signaling cascades regulating hematopoiesis and apoptosis and uptake/degradation of hyaluronic acid. CD44s variant expressed on lymphohematopoietic cell surface, CD44V8–10/CD44E preferentially expressed on ECs, CD44V3–10 expressed in keratinocytes.	[94]
CD90	*THY1*	YES	Mesenchymal stem cells	GPI-anchored glycoprotein. It has diverse functions depending on organ system (neurite growth, cell adhesion, wound repair); counteracts TGFβ1 inhibitory effects on ECs; and has physiological levels of CD105 required for angiogenesis (TGFβ generally regulates pericyte differentiation, adhesion). CD90+ pericytes have higher tendency for proliferation and are more receptive to TGFβ stimulation, after which they undergo cellular senescence/display mature pericyte phenotype (CNS pericytes); CD90– pericytes are more receptive to inflammatory stimulation (IFN–γ, LPS), leading to ICAM1 and MCP1 upregulation in these cells, and leukocyte extravasation in the CNS. Presence of CD90 is one of the defining characteristics of mesenchymal stem cell populations.	[95,96]
CD105	*ENG*	YES	Mesenchymal stem cells	Endoglin, auxiliary receptor for the TGFβ superfamily of proteins. its expression is upregulated in angiogenesis (sprouting edge), with a threshold level required to trigger angiogenesis; it regulates EC migration towards a VEGF stimulus, vascular stability/maturation, and mural cell recruitment to developing vascular tubes. Presence of CD105 is one of the defining characteristics of mesenchymal stem cell populations.	[1,97,98]
CD133	*PROM1*	NO	EPC	Glycosylated transmembrane protein, binds cholesterol, associated with membrane lipid rafts; expression downregulated in mature EC, distinguishes progenitor from mature EC populations. Involved in vesicle trafficking (release of intracellular vesicles), cell membrane protrusions (microvilli, primary cilia and motile cilia, filopodia), regulation of cell signaling pathways (PI3K/AKT implicated in cell self-renewal/tumorigenesis, Src–FAK implicated in cytoskeleton, cell motility/metastasis); CD133 interacts with VEGF, stabilizes dimer formation of VEGF164, promotes tumor growth (regulation of angiogenesis, suppression of cell apoptosis).	[99,100,101]
CD146	*MCAM*	YES	EC, mesenchymal stem cells	Ubiquitously expressed amongst pericytes in most tissues, co-receptor for PDGFRβ in pericytes, PDGF–B/PDGFRβ–mediated pericyte recruitment to assembling vascular tubes (vessel maturation, stability); low specificity for pericytes, populations lacking CD146 have also been identified [102].	[4,102,103]
CD201 (EPCR)	*PROCR*	NO	HSPC, EC (typically large vessels)	Transmembrane protein involved in regulation of coagulation (limits binding of thrombin), inflammation via protein C/activated protein C binding (limits coagulation, inflammation, apoptosis), and contributes to endothelial barrier integrity. Soluble EPCR binds neutrophils, regulates neutrophil adhesion to endothelium.	[104,105,106]
CNN1	*CNN1*	YES	EC, Vascular SMC	Actin filament-associated protein, regulates SMC contraction, involved in the maintenance of the contractile phenotype and differentiation/maturation of SMC. It inhibits myosin ATPase (normally stimulated by actin) and inhibits Ca2+–dependent actin migration; has cytoskeleton stability; and has high CNN1 levels associated with the vascular SMC contractile phenotype. Pericyte CNN1 expression associated with switch towards contractile phenotypes and involvement in aortic aneurysm; endothelial CNN1 expression is associated with aortic aneurysm.	[7,107,108,109]
FGF	*FGF*	YES	EC, Vascular SMC	Growth factor, involved in EC proliferation/migration/differentiation (autocrine), lumenization during vasculogenesis/angiogenesis, and vascular tube maturation/enlargement. Involved in vascular SMC proliferation/migration, vascular tube enlargement, pericyte proliferation/migration, and pericyte recruitment to developing vascular tubes, along with VEGF and PDGF; FGF2 deficiency associated with transition towards the contractile phenotype in pericytes of the aortic adventitia.	[7,110,111]
HLA–DR	*HLA–DRA, HLA–DRB1/B3/B4/B5*	YES	EC	ECs can express HLA–DR during inflammatory states, after induction by pro–inflammatory mediators (IFN–γ, IL–17). Pericytes can also express HLA–DR, participating in antigen presentation along with dendritic cells, contributing to atherosclerosis. Human placental microvascular pericytes can normally activate alloreactive memory T–lymphocytes, though upon IFN–γ stimulation, pericytes suppress memory T-lymphocyte activation. Absence of HLA–DR is one of the defining characteristics of mesenchymal stem cell populations.	[81,112,113]
Nestin	*NES*	YES	Stem/progenitor populations	Class VI IF protein, expressed in the CNS, and during muscle development, contributes to morphogenesis in various organ systems with expression downregulated in mature tissues; generally expressed in stem/progenitor populations. Used to identify pericyte–like cells with stem/progenitor–like qualities in transgenic models of PVAT fibrosis; in these models, pericyte–like cells acquire pro-fibrotic phenotypes, contributing to perivascular fibrosis.	[1,4,50,114,115,116,117]
NG2	*CSPG4*	YES	Vascular SMC (large vessels including aorta), CM	Receptor for various ECM components, including collagen type VI, contributes to mural cell migration during development. It binds/acts as a co-receptor for PDGF–AA and bFGF and contributes to survival/proliferation/migration of mural cells. It binds to plasminogen, facilitating plasminogen activation by plasmin and contributing to TGFβ1 activation.	[1,4,49,118,119,120]
Ninjurin–1	*NINJ1*	YES	EC	Absence of ninjurin–1 protein in vasa vasorum pericytes contributes to microvessel instability and fragility; ninjurin–1 absence also contributes to intimal hyperplasia during vascular injury.	[121]
PDGF–BB	*PDGFB*	YES	EC	Family of polypeptide GF (includes PDGF–A, PDGF–B, PDGF–C, PDGF–D, and PDGF–BB), binds to one of two receptor isoforms, PDGFRα or PDGFRβ; PDGF–BB is the homodimer form of PDGF–B. It is secreted by ECs during angiogenesis. PDGF–BB/PDGFRβ binding on pericytes allows for pericyte recruitment into assembling endothelial vascular tubes, vascular stabilization/maturation, EC proliferation/migration/survival, and muscle cell proliferation/maturation.	[120,122,123,124]
PDGFRβ	*PDGFRB*	YES	Vascular SMC, EC	PDGF represents a family of polypeptide growth factors, including PDGF–A, PDGF–B, PDGF–C, PDGF–D, and PDGF–BB; it binds to one of two receptor isoforms, PDGFRα or PDGFRβ; PDGFRα/β belongs to class III receptor tyrosine kinase family. Pericyte PDGFRβ can bind to EC-secreted PDGF–B, triggering pericyte recruitment to assembling endothelial vascular tubes, facilitating vascular stabilization; EC–pericyte interactions facilitate adhesion/migration/proliferation; PDGF–BB/PDGFRβ signaling is also involved in pericyte survival and development.	[4,120,123,125,126,127]
SCA–1	*LY6A/SCA–1*	NO	CPC	GPI-linked protein that belongs to the family of proteins containing a UPAR domain; UPAR domains are involved in cell migration/adhesion via integrin binding/regulation of integrin expression/function; SCA–1 is involved in stem/progenitor cell lineage fate decisions, regulation of c–Kit expression. SCA–1 expressed in CPC populations that also express cardiac lineage transcription factors such as Mef2, GATA4 and can differentiate into CM/EC/fibroblast groups; SCA–1+ vascular progenitors in the aorta TA contribute to replenishment of the endothelial layer after injury/neointima formation in atherosclerosis (differentiation into EC, vascular SMC populations); SCA–1 expression physiologically low in endothelium, expression increased after injury.	[49,128,129,130]
SM22	*TAGLN*	YES	Vascular SMC (contractile phenotype)	Actin-binding protein. It is associated with actin filaments and the cytoskeleton, modulates signaling pathways in vascular SMCs, affects phenotypic switch in vascular SMCs, and contributes to vascular wall homeostasis; expression is reduced in conditions such as atherosclerosis, neointima formation, aortic aneurysm and dissection. Pericyte SM22 expression associated with switch towards contractile phenotypes, aortic aneurysm progression.	[7,131,132]
Tie2	*TEK*	YES	EC	Binds angiopoietin 1/2, involved in regulation of sprouting angiogenesis both in vitro (sprouting) and in vivo (spheroid assay). ‘Bidirectional model of Tie2/Angiopoietin binding in pericytes and ECs. Pericyte Tie2 binds to EC–derived angiopoietin 2 and EC Tie2 binds to pericyte–derived angiopoietin 1; angiopoietin 1 binding to Tie2 contributes to vascular maturation; angiopoietin 2 contributes to vascular destabilization.	[20]
VEGFR2	*Flk–1* (mouse), VEGFR2, KDR	NO	EC, EPC	Transmembrane receptor tyrosine kinase. In ECs, involved in angiogenesis, including EC survival, proliferation, differentiation and formation of vascular tubes along with regulation of vascular maturation/vascular permeability. Binds VEGFA, VEGFC, and VEGFD, and stimulates signaling pathways comprising many different mediators (PI3K/AKT, ERK1/2, HSP27 signaling). In EPCs, associated with vasculogenesis during early development; inactivation of VEGFR2 during early murine development leads to embryonic death during E8.50–E9.0, owing to failure of vasculogenesis/hematopoietic development. Anti–VEGFR2 therapies can successfully target immature, non-pericyte-coated vessels, but do not affect mature, pericyte-coated vessels.	[133,134,135]
Von Willebrand factor	*VWF*	NO	EC	Glycoprotein involved in coagulation/hemostasis, via mediation of platelet adhesion, during vascular endothelial injury/endothelial activation. Expressed/stored in ECs and megakaryocytes.	[1,136]
Wilm’s tumor protein	*WT1*	YES	Vascular SMC	Transcriptional regulator. Regulates development of tissues derived from the intermediate mesoderm; involved in angiogenesis, vascular SMC proliferation, and vascular remodeling in both physiological and pathophysiological states.	[4,50,137]
α–SMA	*ACTA2*	YES	Vascular SMC	Expressed by pericytes that acquire a smooth muscle phenotype in vitro, particularly after culturing with TGFβ1 and PDGF–BB; target pericytes exhibit either SM–MHC expression, or α–SMA and CNN expression, along with spindle-like cellular morphology. Expressed in CD146+ α–SMA+ pericytes (in vivo), which have been characterized as mature quiescent pericytes in older studies.	[1,4,120]

## Data Availability

No new data were created or analyzed in this study.

## Abbreviations

The following abbreviations are used in this manuscript:

2A7	Alpha–Synuclein antibody
3D	Three–dimensional
3G5	O–Sialoganglioside
AAA	Abdominal aortic aneurysm
ACTA2	Actin alpha 2, Smooth muscle
ADSC	Adipose stromal cell
AKT	Protein kinase B (PKB)
ALP	Alkaline phosphatase
ASC	Adipose stem cell
BAT	Brown adipose tissue
BBB	Blood–brain barrier
BRB	Blood retina barrier
c–Kit	KIT Proto–oncogene, receptor tyrosine kinase
CD11b	ITGAM (integrin subunit Alpha M)
CD14	CD14 molecule
CD19	CD19 molecule
CD31	PECAM1 (platelet and endothelial cell adhesion molecule 1)
CD34	CD34 molecule
CD44	CD44 molecule
CD45	PTPRC (protein tyrosine phosphatase receptor, Type C)
CD56	NCAM1 (neural cell adhesion molecule 1)
CD68	CD68 molecule
CD73	NT5E (5′ Nucleosidase, Ecto)
CD79a	CD79a molecule, immunoglobulin-associated alpha
CD90	THY1 (Thy–1 cell surface antigen)
CD105	ENG (endoglin)
CD133	PROM1 (prominin 1)
CD146	MCAM (melanoma cell adhesion molecule)
CD201	PROCR (protein C receptor)
CNN1	Calponin 1
CNS	Central nervous system
CT	Computed tomography
EC	Endothelial cell
ECM	Extracellular matrix
EEL	External elastic lamina
EGF	Epidermal growth factor
EndMT	Endothelial–to–mesenchymal transition
EPC	Endothelial progenitor cell
ERK	Extracellular signal-related kinase
MAPK1	Mitogen–associated protein kinase 1
FACS	Fluorescence-activated cell sorting
Flk–1	Protein tyrosine kinase receptor Flk–1
KDR	Kinase insert domain receptor
VEGFR2	Vascular endothelial growth factor receptor 2
FGF2 (bFGF)	Fibroblast growth factor 2 (basic fibroblast growth factor)
FGFR	Fibroblast growth factor receptor
GLUT1	Glucose transporter, type 1
HIF–1α	Hypoxia inducible factor–1 alpha
HLA–DR	Human leukocyte antigen, D–related (DR)
IEL	Internal elastic lamina
IL–6	Interleukin-6
ISCT^®^	International Society for Cell & Gene Therapy
LDL	Low–density lipoprotein
lncRNA	Long non–coding RNA
MAPK	Mitogen–associated protein kinase
MEK	Mitogen–associated extracellular signal–regulated kinase
MEMA	Mucoid extracellular matrix (ECM) accumulation
Mt–1α	Metallotheionin–1 alpha
MPC	Mesodermal progenitor cell
MSC	Mesenchymal stromal cell
MYH11	Myosin heavy chain 11
MYL9	Myosin light chain 9
NAT	Nuclear antisense transcript
NG2	Neural glial antigen 2
NG2	Chondroitin sulfate proteoglycan
NUDT6	Nucleoside diphosphate-linked moiety X motif 6
PDGF–BB	Platelet–derived growth factor BB (two B-type subunits)
PDGFRβ	Platelet derived growth factor beta
PF4	Platelet factor 4
PGA	Polyglycolic acid
PGC–1α	Peroxisome proliferator-activated receptor gamma coactivator 1–alpha
PI3K	Phosphoinositide 3 kinase
PIP2	Phosphatidylinositol–(4, 5) biphosphate
PIP3	Phosphatidylinositol–(3, 4, 5) triphosphate
PTEN	Phosphatase and tensin homolog deleted on chromosome 10
PVAT	Perivascular adipose tissue
RNA	Ribonucleic acid
SCA–1	Stem cell antigen–1
LY6A	Lymphocyte antigen 6 complex, locus A
SMC	Smooth muscle cell
TAA	Thoracic aortic aneurysm
TAD	Thoracic aortic dissection
TAGLN	Transgelin
SM22	Smooth muscle protein 22 alpha
TNF–α	Tumor necrosis factor alpha
TSP–1	Thrombospondin–1
TGF–β	Transforming growth factor beta
UCP–1	Uncoupling protein–1 Thermogenin
VEGF	Vascular endothelial growth factor
vWF	von Willebrand factor
WAT	White adipose tissue
α–SMA	alpha-Smooth muscle actin, ACTA2 (actin alpha 2, smooth muscle)

## References

[1] Billaud M., Donnenberg V.S., Ellis B.W., Meyer E.M., Donnenberg A.D., Hill J.C., Richards T.D., Gleason T.G., Phillippi J.A. (2017). Classification and Functional Characterization of Vasa Vasorum-Associated Perivascular Progenitor Cells in Human Aorta. Stem Cell Rep..

[2] Boström K., Watson K.E., Horn S., Wortham C., Herman I.M., Demer L.L. (1993). Bone Morphogenetic Protein Expression in Human Atherosclerotic Lesions. J. Clin. Investig..

[3] Orekhov A.N., Bobryshev Y.V., Chistiakov D.A. (2014). The Complexity of Cell Composition of the Intima of Large Arteries: Focus on Pericyte–like Cells. Cardiovasc. Res..

[4] Crisan M., Yap S., Casteilla L., Chen C.-W., Corselli M., Park T.S., Andriolo G., Sun B., Zheng B., Zhang L. (2008). A Perivascular Origin for Mesenchymal Stem Cells in Multiple Human Organs. Cell Stem Cell.

[5] Faggion Vinholo T., Brownstein A.J., Ziganshin B.A., Zafar M.A., Kuivaniemi H., Body S.C., Bale A.E., Elefteriades J.A. (2019). Genes Associated with Thoracic Aortic Aneurysm and Dissection: 2019 Update and Clinical Implications. Aorta.

[6] Magouliotis D.E., Sicouri S., Sicouri N., Baudo M., Cabrucci F., Yamashita Y., Ramlawi B. (2025). Epigenetic Biomarkers in Thoracic Aortic Aneurysm, Dissection, and Bicuspid Aortopathy: A Comprehensive Review. Biomolecules.

[7] Huang W., Hill J.C., Patel S., Richards T.D., Sultan I., Kaczorowski D.J., Phillippi J.A. (2025). Deficiency of Fibroblast Growth Factor 2 Promotes Contractile Phenotype of Pericytes in Ascending Thoracic Aortic Aneurysm. Am. J. Physiol. Heart Circ. Physiol..

[8] Mohammed K., Avolio E., Mohamed Ahmed E., Rajakaruna C., Ghoneim A., Elminshawy A., Angelini G., Madeddu P. (2025). Exploring Adventitial Pericyte Dysfunction and MEK/ERK Signalling as Therapeutic Targets in Thoracic Aortic Aneurysm. Eur. Heart J..

[9] Armulik A., Genové G., Betsholtz C. (2011). Pericytes: Developmental, Physiological, and Pathological Perspectives, Problems, and Promises. Dev. Cell.

[10] Díaz-Flores L., Gutiérrez R., Madrid J.F., Varela H., Valladares F., Acosta E., Martín-Vasallo P., Díaz-Flores L. (2009). Pericytes. Morphofunction, Interactions and Pathology in a Quiescent and Activated Mesenchymal Cell Niche. Histol. Histopathol..

[11] Dave J.M., Saito J., Mottola G., Greif D.M. (2022). Out to the Tissues: The Arterial Side (Arteries, Arterioles—Development, Structure, Functions, and Pathologies). The Vasculome.

[12] Yamazaki T., Mukouyama Y. (2018). Tissue Specific Origin, Development, and Pathological Perspectives of Pericytes. Front. Cardiovasc. Med..

[13] Cen K., Huang Y., Xie Y., Liu Y. (2024). The Guardian of Intracranial Vessels: Why the Pericyte?. Biomed. Pharmacother..

[14] Geevarghese A., Herman I.M. (2014). Pericyte-Endothelial Crosstalk: Implications and Opportunities for Advanced Cellular Therapies. Transl. Res..

[15] Uemura M.T., Maki T., Ihara M., Lee V.M.Y., Trojanowski J.Q. (2020). Brain Microvascular Pericytes in Vascular Cognitive Impairment and Dementia. Front. Aging Neurosci..

[16] Klaassen I., Van Noorden C.J.F., Schlingemann R.O. (2013). Molecular Basis of the Inner Blood-Retinal Barrier and Its Breakdown in Diabetic Macular Edema and Other Pathological Conditions. Prog. Retin. Eye Res..

[17] Solár P., Zamani A., Lakatosová K., Joukal M. (2022). The Blood–Brain Barrier and the Neurovascular Unit in Subarachnoid Hemorrhage: Molecular Events and Potential Treatments. Fluids Barriers CNS.

[18] Waxman S., Villafranca-Baughman D., Phillippi J., Jakobs T.C., Alarcon-Martinez L., Di Polo A., Sigal I.A. (2025). Pericytes in the Optic Nerve Head. Prog. Retin. Eye Res..

[19] Dabravolski S.A., Markin A.M., Andreeva E.R., Eremin I.I., Orekhov A.N., Melnichenko A.A. (2022). Emerging Role of Pericytes in Therapy of Cardiovascular Diseases. Biomed. Pharmacother..

[20] Teichert M., Milde L., Holm A., Stanicek L., Gengenbacher N., Savant S., Ruckdeschel T., Hasanov Z., Srivastava K., Hu J. (2017). Pericyte-Expressed Tie2 Controls Angiogenesis and Vessel Maturation. Nat. Commun..

[21] Sokolis D.P. (2007). Passive Mechanical Properties and Structure of the Aorta: Segmental Analysis. Acta Physiol..

[22] Burke-Kleinman J., Gotlieb A.I. (2023). Progression of Arterial Vasa Vasorum from Regulator of Arterial Homeostasis to Promoter of Atherogenesis. Am. J. Pathol..

[23] Rolf-Pissarczyk M., Schussnig R., Fries T.-P., Fleischmann D., Elefteriades J.A., Humphrey J.D., Holzapfel G.A. (2025). Mechanisms of Aortic Dissection: From Pathological Changes to Experimental and *in Silico* Models. Prog. Mater. Sci..

[24] Mei C.C., Zhang J., Jing H.X. (2018). Fluid Mechanics of Windkessel Effect. Med. Biol. Eng. Comput..

[25] Marsh J.N., Takiuchi S., Lin S.J., Lanza G.M., Wickline S.A. (2004). Ultrasonic Delineation of Aortic Microstructure: The Relative Contribution of Elastin and Collagen to Aortic Elasticity. J. Acoust. Soc. Am..

[26] Astrand H., Stalhand J., Karlsson J., Karlsson M., Sonesson B., Länne T. (2011). In Vivo Estimation of the Contribution of Elastin and Collagen to the Mechanical Properties in the Human Abdominal Aorta: Effect of Age and Sex. J. Appl. Physiol..

[27] Singh M., Ziganshin B.A., Elefteriades J.A., Vasan R.S., Sawyer D.B. (2018). Aortic Aneurysm. Encyclopedia of Cardiovascular Research and Medicine.

[28] Grewal N., Dolmaci O., Klautz A., Legue J., Driessen A., Klautz R., Poelmann R. (2023). The Role of Transforming Growth Factor Beta in Bicuspid Aortic Valve Aortopathy. Indian J. Thorac. Cardiovasc. Surg..

[29] Xiong W., Zhao X., Villacorta L., Rom O., Garcia-Barrio M.T., Guo Y., Fan Y., Zhu T., Zhang J., Zeng R. (2018). Brown Adipocyte-Specific PPARγ (Peroxisome Proliferator-Activated Receptor γ) Deletion Impairs Perivascular Adipose Tissue Development and Enhances Atherosclerosis in Mice. Arterioscler. Thromb. Vasc. Biol..

[30] Ueda K. (2026). Perivascular Adipose Tissue in Cardiovascular Disease: From Mechanisms to Therapeutic Targets. J. Cardiol..

[31] Shi K., Anmin R., Cai J., Qi Y., Han W., Li M., Zhang G., Zhang S., Fu L., Han W. (2022). Ascending Aortic Perivascular Adipose Tissue Inflammation Associates with Aortic Valve Disease. J. Cardiol..

[32] Cai M., Zhao D., Han X., Han S., Zhang W., Zang Z., Gai C., Rong R., Gao T. (2023). The Role of Perivascular Adipose Tissue-Secreted Adipocytokines in Cardiovascular Disease. Front. Immunol..

[33] Majesky M.W. (2015). Adventitia and Perivascular Cells. Arter. Thromb. Vasc. Biol..

[34] Virmani R., Sato Y., Romero M.E., Butany J. (2022). Aneurysms of the Aorta: Ascending, Thoracic, and Abdominal and Their Management. Cardiovascular Pathology.

[35] Tonar Z., Tomášek P., Loskot P., Janáček J., Králíčková M., Witter K. (2016). Vasa Vasorum in the Tunica Media and Tunica Adventitia of the Porcine Aorta. Ann. Anat.-Anat. Anz..

[36] Billaud M., Hill J.C., Richards T.D., Gleason T.G., Phillippi J.A. (2018). Medial Hypoxia and Adventitial Vasa Vasorum Remodeling in Human Ascending Aortic Aneurysm. Front. Cardiovasc. Med..

[37] Hu Y., Xu Q. (2011). Adventitial Biology. Arterioscler. Thromb. Vasc. Biol..

[38] Weis-Müller B.T., Modlich O., Drobinskaya I., Unay D., Huber R., Bojar H., Schipke J.D., Feindt P., Gams E., Müller W. (2006). Gene Expression in Acute Stanford Type A Dissection: A Comparative Microarray Study. J. Transl. Med..

[39] Suguro T., Watanabe T., Ban Y., Kodate S., Misaki A., Hirano T., Miyazaki A., Adachi M. (2007). Increased Human Urotensin II Levels Are Correlated with Carotid Atherosclerosis in Essential Hypertension. Am. J. Hypertens..

[40] Chai S.B., Li X.M., Pang Y.Z., Qi Y.F., Tang C.S. (2010). Increased Plasma Levels of Endothelin-1 and Urotensin-II in Patients with Coronary Heart Disease. Heart Vessel..

[41] Gruson D., Rousseau M.F., Ahn S.A., van Linden F., Ketelslegers J.M. (2006). Circulating Urotensin II Levels in Moderate to Severe Congestive Heart Failure: Its Relations with Myocardial Function and Well Established Neurohormonal Markers. Peptides.

[42] Zhang Y.-G., Kuang Z.-J., Mao Y.-Y., Wei R.-H., Bao S.-L., Wu L.-B., Li Y.-G., Tang C.-S. (2011). Osteopontin Is Involved in Urotensin II-Induced Migration of Rat Aortic Adventitial Fibroblasts. Peptides.

[43] Barbour J.R., Spinale F.G., Ikonomidis J.S. (2007). Proteinase Systems and Thoracic Aortic Aneurysm Progression. J. Surg. Res..

[44] Viswanathan S., Shi Y., Galipeau J., Krampera M., Leblanc K., Martin I., Nolta J., Phinney D.G., Sensebe L. (2019). Mesenchymal Stem versus Stromal Cells: International Society for Cell & Gene Therapy (ISCT®) Mesenchymal Stromal Cell Committee Position Statement on Nomenclature. Cytotherapy.

[45] Medici D., Shore E.M., Lounev V.Y., Kaplan F.S., Kalluri R., Olsen B.R. (2010). Conversion of Vascular Endothelial Cells into Multipotent Stem-like Cells. Nat. Med..

[46] Zimmerlin L., Donnenberg V.S., Pfeifer M.E., Meyer E.M., Péault B., Rubin J.P., Donnenberg A.D. (2009). Stromal Vascular Progenitors in Adult Human Adipose Tissue. Cytom. Part A.

[47] Wang Y., Thottappillil N., Gomez-Salazar M., Tower R.J., Qin Q., Del Rosario Alvia I.C., Xu M., Cherief M., Cheng R., Archer M. (2024). Integrated Transcriptomics of Human Blood Vessels Defines a Spatially Controlled Niche for Early Mesenchymal Progenitor Cells. Dev. Cell.

[48] Ferrante A., Guggino G., Di Liberto D., Ciccia F., Cipriani P., Balistreri C.R., Sireci G., Giacomelli R., Triolo G. (2016). Endothelial Progenitor Cells: Are They Displaying a Function in Autoimmune Disorders?. Mech. Ageing Dev..

[49] Zhang L., Issa Bhaloo S., Chen T., Zhou B., Xu Q. (2018). Role of Resident Stem Cells in Vessel Formation and Arteriosclerosis. Circ. Res..

[50] Vasuri F., Fittipaldi S., Buzzi M., Degiovanni A., Stella A., D’Errico-Grigioni A., Pasquinelli G. (2012). Nestin and WT1 Expression in Small–sized Vasa Vasorum from Human Normal Arteries. Histol. Histopathol..

[51] Chen C.-W., Okada M., Proto J.D., Gao X., Sekiya N., Beckman S.A., Corselli M., Crisan M., Saparov A., Tobita K. (2013). Human Pericytes for Ischemic Heart Repair. Stem Cells.

[52] West C.C., Hardy W.R., Murray I.R., James A.W., Corselli M., Pang S., Black C., Lobo S.E., Sukhija K., Liang P. (2016). Prospective Purification of Perivascular Presumptive Mesenchymal Stem Cells from Human Adipose Tissue: Process Optimization and Cell Population Metrics across a Large Cohort of Diverse Demographics. Stem Cell Res. Ther..

[53] Petrini M., Pacini S., Trombi L., Fazzi R., Montali M., Ikehara S., Abraham N.G. (2009). Identification and Purification of Mesodermal Progenitor Cells from Human Adult Bone Marrow. Stem Cells Dev..

[54] Scott S.S., Yang X., Robich M., Liaw L., Boucher J.M. (2019). Differentiation Capacity of Human Aortic Perivascular Adipose Progenitor Cells. J. Vis. Exp..

[55] Gimble J.M., Bunnell B.A., Frazier T., Rowan B., Shah F., Thomas-Porch C., Wu X. (2013). Adipose-Derived Stromal/Stem Cells. Organogenesis.

[56] Baer P.C. (2014). Adipose-Derived Mesenchymal Stromal/Stem Cells: An Update on Their Phenotype in Vivo and in Vitro. World J. Stem Cells.

[57] Avolio E., Madeddu P. (2016). Discovering Cardiac Pericyte Biology: From Physiopathological Mechanisms to Potential Therapeutic Applications in Ischemic Heart Disease. Vasc. Pharmacol..

[58] Beltrami A.P., Madeddu P. (2018). Pericytes and Cardiac Stem Cells: Common Features and Peculiarities. Pharmacol. Res..

[59] Thottapillil N., Corselli M., Murray I., Hardy R., Gomez-Salazar M., Casamitjana J., Shaw I., Wang Z., Vezzani B., Ding L. (2025). Mesenchymal Progenitor Cells in Perivascular Niches: Forerunners of Mesenchymal Stem Cells and Players in Tissue Scarring and Regeneration. Vasc. Pharmacol..

[60] Corselli M., Chin C.J., Parekh C., Sahaghian A., Wang W., Ge S., Evseenko D., Wang X., Montelatici E., Lazzari L. (2013). Perivascular Support of Human Hematopoietic Stem/Progenitor Cells. Blood.

[61] Kabara M., Kawabe J., Matsuki M., Hira Y., Minoshima A., Shimamura K., Yamauchi A., Aonuma T., Nishimura M., Saito Y. (2014). Immortalized Multipotent Pericytes Derived from the Vasa Vasorum in the Injured Vasculature. A Cellular Tool for Studies of Vascular Remodeling and Regeneration. Lab. Investig..

[62] Wiegreffe C., Christ B., Huang R., Scaal M. (2009). Remodeling of Aortic Smooth Muscle during Avian Embryonic Development. Dev. Dyn..

[63] Pouget C., Pottin K., Jaffredo T. (2008). Sclerotomal Origin of Vascular Smooth Muscle Cells and Pericytes in the Embryo. Dev. Biol..

[64] Stratman A.N., Pezoa S.A., Farrelly O.M., Castranova D., Dye L.E., Butler M.G., Sidik H., Talbot W.S., Weinstein B.M. (2017). Interactions between Mural Cells and Endothelial Cells Stabilize the Developing Zebrafish Dorsal Aorta. Development.

[65] Gerhardt H., Betsholtz C. (2003). Endothelial-Pericyte Interactions in Angiogenesis. Cell Tissue Res..

[66] Santoro M.M., Pesce G., Stainier D.Y. (2009). Characterization of Vascular Mural Cells during Zebrafish Development. Mech. Dev..

[67] Halper J. (2018). Basic Components of Vascular Connective Tissue and Extracellular Matrix. Advances in Pharmacology.

[68] Stępień K.L., Auguściak-Duma A., Fus-Kujawa A., Diak N., Lesiak M., Bogunia E., Hermyt M., Czekaj P., Sznapka M., Ziaja D. (2026). Abdominal Aortic Aneurysm: Characteristics of Extracellular Matrix Abnormalities Caused by Disorders of Collagen Types I and III and Elastin. Int. J. Cardiol..

[69] Bjorgvinsdottir O., Ferguson S.J., Snorradottir B.S., Gudjonsson T., Wuertz-Kozak K. (2024). The Influence of Physical and Spatial Substrate Characteristics on Endothelial Cells. Mater. Today Bio.

[70] Gui C., Chang W., Liu Y., Tao L., Shen D., Ge M. (2025). Modeling and Experimental Research of Heat and Mass Transfer during the Freeze-Drying of Porcine Aorta Considering Radially-Layered Tissue Properties. Front. Heat Mass Transf..

[71] Jana S., Hu M., Shen M., Kassiri Z. (2019). Extracellular Matrix, Regional Heterogeneity of the Aorta, and Aortic Aneurysm. Exp. Mol. Med..

[72] Kielty C.M., Bax D.V., Hodson N., Sherratt M.J. (2005). Extracellular Matrix Molecules in Vascular Tissue Engineering. Surfaces and Interfaces for Biomaterials.

[73] Zafar M.A., Peterss S., Ziganshin B.A., Elefteriades J.A. (2018). Histology of Aortic Disease and Progression of Aortic Dissection from Acute to Chronic. New Approaches to Aortic Diseases from Valve to Abdominal Bifurcation.

[74] Andreeva E.R., Pugach I.M., Gordon D., Orekhov A.N. (1998). Continuous Subendothelial Network Formed by Pericyte–like Cells in Human Vascular Bed. Tissue Cell.

[75] Andreeva E.R., Rekhter M.D., Romanov Y.A., Antonova G.M., Antonov A.S., Mironov A.A., Orekhov A.N. (1992). Stellate Cells of Aortic Intima: II. Arborization of Intimal Cells in Culture. Tissue Cell.

[76] Andreeva E.R., Pugach I.M., Orekhov A.N. (1997). Subendothelial Smooth Muscle Cells of Human Aorta Express Macrophage Antigen in Situ and in Vitro. Atherosclerosis.

[77] Van Camp L., Vanhooren J., Depreter B., Hofmans M., D’Hont I., Chantrain C., Dedeken L., Van Damme A., Uyttebroeck A., Lammens T. (2024). Deciphering the Role and Therapeutic Potential of Macrophage Marker CD68 in Pediatric Acute Myeloid Leukemia. Blood.

[78] Rekhter M.D., Andreeva E.R., Mironov A.A., Orekhov A.N. (1991). Three-Dimensional Cytoarchitecture of Normal and Atherosclerotic Intima of Human Aorta. Am. J. Pathol..

[79] Andreeva E.R., Serebryakov V.N., Orekhov A.N. (1995). Gap Junctional Communication in Primary Culture of Cells Derived from Human Aortic Intima. Tissue Cell.

[80] Orekhov A.N., Bobryshev Y.V., Orekhov A.N., Bobryshev Y.V. (2015). Cell Composition of the Subendothelial Aortic Intima and the Role of Alpha-Smooth Muscle Actin Expressing Pericyte–like Cells and Smooth Muscle Cells in the Development of Atherosclerosis. Muscle Cell and Tissue.

[81] Ivanova E.A., Orekhov A.N. (2016). Cellular Model of Atherogenesis Based on Pluripotent Vascular Wall Pericytes. Stem Cells Int..

[82] Nayak R.C., Berman A.B., George K.L., Eisenbarth G.S., King G.L. (1988). A Monoclonal Antibody (3G5)-Defined Ganglioside Antigen Is Expressed on the Cell Surface of Microvascular Pericytes. J. Exp. Med..

[83] Orekhov A.N., Andreeva E.R. (2025). Intimal Macrovascular Pericytes: Their Role in Vascular Biology and Atherogenesis. Curr. Med. Chem..

[84] Fancello I., Willett S., Castiglioni C., Amer S., Santoleri S., Bragg L., Galli F., Cossu G. (2025). TNAP Expressing Adventitial Pericytes Contribute to Myogenesis during Foetal Development. Vasc. Pharmacol..

[85] Makris K., Mousa C., Cavalier E. (2023). Alkaline Phosphatases: Biochemistry, Functions, and Measurement. Calcif. Tissue Int..

[86] Rogers M.S., D’Amato R.J. (2006). The Effect of Genetic Diversity on Angiogenesis. Exp. Cell Res..

[87] Afonyushkin T., Oskolkova O.V., Bochkov V.N. (2018). Oxidized Phospholipids Stimulate Production of Stem Cell Factor via NRF2-Dependent Mechanisms. Angiogenesis.

[88] Du J., Song J., Ding L., Fan X., Lin L., Li A., Liang L., Kong X. (2023). Treatment with Imatinib Was Useful to Delay the Neointimal Hyperplasia of Aortocaval Fistula in Adenine-Induced Renal Failure Rats. Biochem. Biophys. Res. Commun..

[89] Gritz E., Hirschi K.K. (2016). Specification and Function of Hemogenic Endothelium during Embryogenesis. Cell. Mol. Life Sci..

[90] Yoon J.W., Jang I.H., Heo S.C., Kwon Y.W., Choi E.J., Bae K.-H., Suh D.-S., Kim S.-C., Han S., Haam S. (2015). Isolation of Foreign Material-Free Endothelial Progenitor Cells Using CD31 Aptamer and Therapeutic Application for Ischemic Injury. PLoS ONE.

[91] Boss A.L., Damani T., Wickman T.J., Chamley L.W., James J.L., Brooks A.E. (2022). Full Spectrum Flow Cytometry Reveals Mesenchymal Heterogeneity in First Trimester Placentae and Phenotypic Convergence in Culture, Providing Insight into the Origins of Placental Mesenchymal Stromal Cells. eLife.

[92] Marvasti T.B., Alibhai F.J., Weisel R.D., Li R.-K. (2019). CD34+ Stem Cells: Promising Roles in Cardiac Repair and Regeneration. Can. J. Cardiol..

[93] Rakocevic J., Orlic D., Mitrovic-Ajtic O., Tomasevic M., Dobric M., Zlatic N., Milasinovic D., Stankovic G., Ostojić M., Labudovic-Borovic M. (2017). Endothelial Cell Markers from Clinician’s Perspective. Exp. Mol. Pathol..

[94] Naor D., Sionov R.V., Ish-Shalom D. (1997). CD44: Structure, Function, and Association with the Malignant Process. Adv. Cancer Res..

[95] Li C., Hampson I.N., Hampson L., Kumar P., Bernabeu C., Kumar S. (2000). CD105 Antagonizes the Inhibitory Signaling of Transforming Growth Factor Beta1 on Human Vascular Endothelial Cells. FASEB J..

[96] Park T.I.-H., Feisst V., Brooks A.E.S., Rustenhoven J., Monzo H.J., Feng S.X., Mee E.W., Bergin P.S., Oldfield R., Graham E.S. (2016). Cultured Pericytes from Human Brain Show Phenotypic and Functional Differences Associated with Differential CD90 Expression. Sci. Rep..

[97] Mancini M.L., Terzic A., Conley B.A., Oxburgh L.H., Nicola T., Vary C.P.H. (2009). Endoglin Plays Distinct Roles in Vascular Smooth Muscle Cell Recruitment and Regulation of Arteriovenous Identity during Angiogenesis. Dev. Dyn..

[98] Ollauri-Ibáñez C., Núñez-Gómez E., Egido-Turrión C., Silva-Sousa L., Díaz-Rodríguez E., Rodríguez-Barbero A., López-Novoa J.M., Pericacho M. (2020). Continuous Endoglin (CD105) Overexpression Disrupts Angiogenesis and Facilitates Tumor Cell Metastasis. Angiogenesis.

[99] Adini A., Adini I., Ghosh K., Benny O., Pravda E., Hu R., Luyindula D., D’Amato R.J. (2013). The Stem Cell Marker Prominin-1/CD133 Interacts with Vascular Endothelial Growth Factor and Potentiates Its Action. Angiogenesis.

[100] Bailey A.S., Fleming W.H. (2003). Converging Roads: Evidence for an Adult Hemangioblast. Exp. Hematol..

[101] Pleskač P., Fargeas C.A., Veselska R., Corbeil D., Skoda J. (2024). Emerging Roles of Prominin-1 (CD133) in the Dynamics of Plasma Membrane Architecture and Cell Signaling Pathways in Health and Disease. Cell. Mol. Biol. Lett..

[102] Tuleta I., Frangogiannis N.G. (2025). Pericytes in Tissue Fibrosis. Am. J. Physiol. Cell Physiol..

[103] Richards A., Khalil A., Bisht P., Whitfield T.W., Gao X., Mooney D., Gehrke L., Jaenisch R. (2026). A Human Blood-Brain Barrier Model Reveals Pericytes as Critical Regulators of Viral Neuroinvasion. iScience.

[104] Esmon C.T. (2006). The Endothelial Protein C Receptor. Curr. Opin. Hematol..

[105] Fink K., Busch H.-J., Bourgeois N., Schwarz M., Wolf D., Zirlik A., Peter K., Bode C., Muhlen C. (2013). von zur Mac-1 Directly Binds to the Endothelial Protein C-Receptor: A Link between the Protein C Anticoagulant Pathway and Inflammation?. PLoS ONE.

[106] Lattenist L., Kers J., Claessen N., Berge I.J.M.t., Bemelman F.J., Florquin S., Roelofs J.J.T.H. (2013). Renal and Urinary Levels of Endothelial Protein C Receptor Correlate with Acute Renal Allograft Rejection. PLoS ONE.

[107] Zhou H., Ke J., Liu C., Zhu M., Xiao B., Wang Q., Hou R., Zheng Y., Wu Y., Zhou X. (2023). Potential Prognostic and Immunotherapeutic Value of Calponin 1: A Pan-Cancer Analysis. Front. Pharmacol..

[108] Yao G., Zheng X., Hu Y., Zhao Y., Kong B., Ti Y., Bu P.L. (2024). FBLN7 Mediates Vascular Smooth Muscle Cell Phenotype Switching and Vascular Remodeling in Hypertension. Theranostics.

[109] Lesiak M., Augusciak-Duma A., Stepien K.L., Fus-Kujawa A., Botor M., Sieron A.L. (2021). Searching for New Molecular Markers for Cells Obtained from Abdominal Aortic Aneurysm. J. Appl. Genet..

[110] Rajasekar J., Perumal M.K., Vallikannan B. (2019). A Critical Review on Anti-Angiogenic Property of Phytochemicals. J. Nutr. Biochem..

[111] Dębski T., Kurzyk A., Ostrowska B., Wysocki J., Jaroszewicz J., Święszkowski W., Pojda Z. (2020). Scaffold Vascularization Method Using an Adipose-Derived Stem Cell (ASC)-Seeded Scaffold Prefabricated with a Flow-through Pedicle. Stem Cell Res. Ther..

[112] Liu R., Merola J., Manes T.D., Qin L., Tietjen G.T., López-Giráldez F., Broecker V., Fang C., Xie C., Chen P.-M. (2018). Interferon-γ Converts Human Microvascular Pericytes into Negative Regulators of Alloimmunity through Induction of Indoleamine 2,3-Dioxygenase 1. JCI Insight.

[113] Zierer J., Cojean C., Osinga R., Läuchli S., Wieczorek G., Roth L., Roediger B. (2025). Hidradenitis Suppurativa Is an HLA-Associated Autoimmune Disease. J. Investig. Dermatol..

[114] Iwayama T., Steele C., Yao L., Dozmorov M.G., Karamichos D., Wren J.D., Olson L.E. (2015). PDGFRα Signaling Drives Adipose Tissue Fibrosis by Targeting Progenitor Cell Plasticity. Genes. Dev..

[115] Liu Y., Xiao F., Hu X., Tang Z., Fu Z., Liang X., Zeng G., Zeng W., Liao Y., Ren Y. (2020). Recovery and Maintenance of NESTIN Expression in Umbilical Cord-MSC Using a Novel Culture Medium. AMB. Express.

[116] Struys T., Krage T., Vandenabeele F., Raab W.H.-M., Lambrichts I. (2005). Immunohistochemical Evidence for Proteolipid Protein and Nestin Expression in the Late Bell Stage of Developing Rodent Teeth. Arch. Oral Biol..

[117] Takamori Y., Mori T., Wakabayashi T., Nagasaka Y., Matsuzaki T., Yamada H. (2009). Nestin-Positive Microglia in Adult Rat Cerebral Cortex. Brain Res..

[118] Murfee W.L., Skalak T.C., Peirce S.M. (2005). Differential Arterial/Venous Expression of NG2 Proteoglycan in Perivascular Cells Along Microvessels: Identifying a Venule-Specific Phenotype. Microcirculation.

[119] Nayak T., Trotter J., Sakry D. (2018). The Intracellular Cleavage Product of the NG2 Proteoglycan Modulates Translation and Cell-Cycle Kinetics via Effects on mTORC1/FMRP Signaling. Front. Cell. Neurosci..

[120] Ozerdem U., Grako K.A., Dahlin-Huppe K., Monosov E., Stallcup W.B. (2001). NG2 Proteoglycan Is Expressed Exclusively by Mural Cells during Vascular Morphogenesis. Dev. Dyn..

[121] Horiuchi K., Kano K., Minoshima A., Hayasaka T., Yamauchi A., Tatsukawa T., Matsuo R., Yoshida Y., Tomita Y., Kabara M. (2021). Pericyte-Specific Deletion of Ninjurin–1 Induces Fragile Vasa Vasorum Formation and Enhances Intimal Hyperplasia of Injured Vasculature. Am. J. Physiol. Heart Circ. Physiol..

[122] Hamaguchi H., Dohi K., Sakai T., Taoka M., Isobe T., Matsui T.S., Deguchi S., Furuichi Y., Fujii N.L., Manabe Y. (2023). PDGF–B Secreted from Skeletal Muscle Enhances Myoblast Proliferation and Myotube Maturation via Activation of the PDGFR Signaling Cascade. Biochem. Biophys. Res. Commun..

[123] Magnusson P.U., Looman C., Åhgren A., Wu Y., Claesson-Welsh L., Heuchel R.L. (2007). Platelet-Derived Growth Factor Receptor-β Constitutive Activity Promotes Angiogenesis In Vivo and In Vitro. Arterioscler. Thromb. Vasc. Biol..

[124] Sheng X., Zhang C., Zhao J., Xu J., Zhang P., Ding Q., Zhang J. (2024). Microvascular Destabilization and Intricated Network of the Cytokines in Diabetic Retinopathy: From the Perspective of Cellular and Molecular Components. Cell Biosci..

[125] Barron L., Gharib S.A., Duffield J.S. (2016). Lung Pericytes and Resident Fibroblasts: Busy Multitaskers. Am. J. Pathol..

[126] Caporali A., Martello A., Miscianinov V., Maselli D., Vono R., Spinetti G. (2017). Contribution of Pericyte Paracrine Regulation of the Endothelium to Angiogenesis. Pharmacol. Ther..

[127] Mao Y., Liu X., Song Y., Zhai C., Zhang L. (2018). VEGF-A/VEGFR-2 and FGF-2/FGFR-1 but Not PDGF–BB/PDGFR-β Play Important Roles in Promoting Immature and Inflammatory Intraplaque Angiogenesis. PLoS ONE.

[128] Bradfute S.B., Graubert T.A., Goodell M.A. (2005). Roles of SCA–1 in Hematopoietic Stem/Progenitor Cell Function. Exp. Hematol..

[129] Ni Z., Lyu L., Gong H., Du L., Wen Z., Jiang H., Yang H., Hu Y., Zhang B., Xu Q. (2023). Multilineage Commitment of SCA–1^+^ Cells in Reshaping Vein Grafts. Theranostics.

[130] Valente M., Nascimento D.S., Cumano A., Pinto-do-Ó P. (2014). SCA–1+ Cardiac Progenitor Cells and Heart-Making: A Critical Synopsis. Stem Cells Dev..

[131] Zhong L., He X., Si X., Wang H., Li B., Hu Y., Li M., Chen X., Liao W., Liao Y. (2019). SM22α (Smooth Muscle 22α) Prevents Aortic Aneurysm Formation by Inhibiting Smooth Muscle Cell Phenotypic Switching Through Suppressing Reactive Oxygen Species/NF–κB (Nuclear Factor-κB). Arterioscler. Thromb. Vasc. Biol..

[132] Sun Y., Zhao Z., Hou L., Xiao Y., Qin F., Yan J., Zhou J., Jing Z. (2017). The Regulatory Role of Smooth Muscle 22 on the Proliferation of Aortic Smooth Muscle Cells Participates in the Development of Aortic Dissection. J. Vasc. Surg..

[133] Barlak N., Sanli F., Celik S., İskender B., Anil D.A., Gambacorta N., Burmaoglu S., Nicolotti O., Algul O., Karatas O.F. (2025). Synthesis and Evaluation of Sulfonamide-Chalcone Hybrid Compounds as Inhibitors of VEGFR1/VEGFR2-Mediated Angiogenesis. Bioorg. Chem..

[134] Benjamin L.E., Hemo I., Keshet E. (1998). A Plasticity Window for Blood Vessel Remodelling Is Defined by Pericyte Coverage of the Preformed Endothelial Network and Is Regulated by PDGF–B and VEGF. Development.

[135] Shibuya M., Claesson-Welsh L. (2006). Signal Transduction by VEGF Receptors in Regulation of Angiogenesis and Lymphangiogenesis. Exp. Cell Res..

[136] Santos-Gomes J., Gandra I., Adão R., Perros F., Brás-Silva C. (2022). An Overview of Circulating Pulmonary Arterial Hypertension Biomarkers. Front. Cardiovasc. Med..

[137] O’Malley D.P., Kim Y.S., Weiss L.M. (2015). Distinctive Immunohistochemical Staining in Littoral Cell Angioma Using ERG and WT-1. Ann. Diagn. Pathol..

[138] Pinard A., Jones G.T., Milewicz D.M. (2019). Genetics of Thoracic and Abdominal Aortic Diseases. Circ. Res..

[139] Tinajero M.G., Gotlieb A.I. (2020). Recent Developments in Vascular Adventitial Pathobiology: The Dynamic Adventitia as a Complex Regulator of Vascular Disease. Am. J. Pathol..

[140] Choi J.Y., Shin M.-Y., Suh S.H., Park S. (2015). Lyso-Globotriaosylceramide Downregulates KCa3.1 Channel Expression to Inhibit Collagen Synthesis in Fibroblasts. Biochem. Biophys. Res. Commun..

[141] Fercana G.R., Yerneni S., Billaud M., Hill J.C., VanRyzin P., Richards T.D., Sicari B.M., Johnson S.A., Badylak S.F., Campbell P.G. (2017). Perivascular Extracellular Matrix Hydrogels Mimic Native Matrix Microarchitecture and Promote Angiogenesis via Basic Fibroblast Growth Factor. Biomaterials.

[142] Mohammed K.A.K., Madeddu P., Avolio E. (2024). MEK Inhibitors: A Promising Targeted Therapy for Cardiovascular Disease. Front. Cardiovasc. Med..

[143] Olszewska-Pazdrak B., Hein T.W., Olszewska P., Carney D.H. (2009). Chronic Hypoxia Attenuates VEGF Signaling and Angiogenic Responses by Downregulation of KDR in Human Endothelial Cells. Am. J. Physiol. Cell Physiol..

[144] Kessler K., Borges L.F., Ho-Tin-Noé B., Jondeau G., Michel J.-B., Vranckx R. (2014). Angiogenesis and Remodelling in Human Thoracic Aortic Aneurysms. Cardiovasc. Res..

[145] Aviles R.J., Annex B.H., Lederman R.J. (2003). Testing Clinical Therapeutic Angiogenesis Using Basic Fibroblast Growth Factor (FGF-2). Br. J. Pharmacol..

[146] Ji J., Xu F., Li L., Chen R., Wang J., Hu W. (2010). Activation of Adventitial Fibroblasts in the Early Stage of the Aortic Transplant Vasculopathy in Rat. Transplantation.

[147] Chen H., Du X. (2024). Increased FGF2 Expression Promotes Immune Cell Infiltration and Correlates with an Unfavorable Prognosis in Thyroid Cancer. Heliyon.

[148] Dessalles C.A., Babataheri A., Barakat A.I. (2021). Pericyte Mechanics and Mechanobiology. J. Cell Sci..

[149] Angouras D., Sokolis D.P., Dosios T., Kostomitsopoulos N., Boudoulas H., Skalkeas G., Karayannacos P.E. (2000). Effect of Impaired Vasa Vasorum Flow on the Structure and Mechanics of the Thoracic Aorta: Implications for the Pathogenesis of Aortic Dissection. Eur. J. Cardiothorac. Surg..

[150] Traktuev D.O., Merfeld-Clauss S., Li J., Kolonin M., Arap W., Pasqualini R., Johnstone B.H., March K.L. (2008). A Population of Multipotent CD34-Positive Adipose Stromal Cells Share Pericyte and Mesenchymal Surface Markers, Reside in a Periendothelial Location, and Stabilize Endothelial Networks. Circ. Res..

[151] Karar J., Maity A. (2011). PI3K/AKT/mTOR Pathway in Angiogenesis. Front. Mol. Neurosci..

[152] Song M., Finley S.D. (2018). Mechanistic Insight into Activation of MAPK Signaling by Pro-Angiogenic Factors. BMC Syst. Biol..

[153] Wu H., Xie C., Wang R., Cheng J., Xu Q., Zhao H. (2023). Comparative Analysis of Thoracic and Abdominal Aortic Aneurysms across the Segment and Species at the Single–cell Level. Front. Pharmacol..

[154] Avolio E., Katare R., Thomas A.C., Caporali A., Schwenke D., Carrabba M., Meloni M., Caputo M., Madeddu P. (2022). Cardiac Pericyte Reprogramming by MEK Inhibition Promotes Arteriologenesis and Angiogenesis of the Ischemic Heart. J. Clin. Investig..

[155] Campagnolo P., Cesselli D., Al Haj Zen A., Beltrami A.P., Kränkel N., Katare R., Angelini G., Emanueli C., Madeddu P. (2010). Human Adult Vena Saphena Contains Perivascular Progenitor Cells Endowed with Clonogenic and Proangiogenic Potential. Circulation.

[156] Kajimoto M., Shimono T., Hirano K., Miyake Y., Sawada Y., Kato N., Hirata H., Imanaka-Yoshida K., Nishikawa M., Yoshida T. (2006). Development of a New Method for Endovascular Aortic Repair. Circulation.

[157] Fujiwara H., Oda K., Saiki Y., Sakamoto N., Ohashi T., Sato M., Tabata Y., Tabayashi K. (2006). The Wrapping Method Using Biodegradable Felt Strips Has a Preventive Effect on the Thinning of the Aortic Wall: Experimental Study in the Canine Aorta. J. Vasc. Surg..

[158] Sato M., Kawamoto S., Watanabe M., Sakamoto N., Sato M., Tabata Y., Saiki Y. (2012). Medial Regeneration Using a Biodegradable Felt as a Scaffold Preserves Integrity and Compliance of a Canine Dissected Aorta. Circulation.

[159] Faghihi M.A., Wahlestedt C. (2009). Regulatory Roles of Natural Antisense Transcripts. Nat. Rev. Mol. Cell Biol..

[160] Winter H., Winski G., Busch A., Chernogubova E., Fasolo F., Wu Z., Bäcklund A., Khomtchouk B.B., Van Booven D.J., Sachs N. (2023). Targeting Long Non-Coding RNA NUDT6 Enhances Smooth Muscle Cell Survival and Limits Vascular Disease Progression. Mol. Ther..

[161] Wang J., He C., Chen Y., Hu X., Xu H., Liu J., Yang Y., Chen L., Li T., Fang L. (2024). Platelet Factors Ameliorate Thoracic Aortic Aneurysm and Dissection by Inhibiting the FGF-FGFR Cascade Activation in Aortic-Endothelial Cell. iScience.

